# Small Molecule c-KIT Inhibitors for the Treatment of Gastrointestinal Stromal Tumors: A Review on Synthesis, Design Strategies, and Structure–Activity Relationship (SAR)

**DOI:** 10.3390/ijms24119450

**Published:** 2023-05-29

**Authors:** Sreenivasulu Godesi, Joohan Lee, Hossam Nada, Guofeng Quan, Ahmed Elkamhawy, Yongseok Choi, Kyeong Lee

**Affiliations:** 1 BK21 FOUR Team and Integrated Research Institute for Drug Development, College of Pharmacy, Dongguk University-Seoul, Goyang 10326, Republic of Korea; 2 Department of Pharmaceutical Organic Chemistry, Faculty of Pharmacy, Mansoura University, Mansoura 35516, Egypt; 3 College of Biosciences and Biotechnology, Korea University, Seoul 02841, Republic of Korea

**Keywords:** c-KIT, GISTs, stem cell growth factor, c-KIT inhibitors, SAR, SCFR

## Abstract

The proto-oncogenic protein, c-KIT, plays a crucial role in regulating cellular transformation and differentiation processes, such as proliferation, survival, adhesion, and chemotaxis. The overexpression of, and mutations, in c-KIT can lead to its dysregulation and promote various human cancers, particularly gastrointestinal stromal tumors (GISTs); approximately 80–85% of cases are associated with oncogenic mutations in the *KIT* gene. Inhibition of c-KIT has emerged as a promising therapeutic target for GISTs. However, the currently approved drugs are associated with resistance and significant side effects, highlighting the urgent need to develop highly selective c-KIT inhibitors that are not affected by these mutations for GISTs. Herein, the recent research efforts in medicinal chemistry aimed at developing potent small-molecule c-KIT inhibitors with high kinase selectivity for GISTs are discussed from a structure–activity relationship perspective. Moreover, the synthetic pathways, pharmacokinetic properties, and binding patterns of the inhibitors are also discussed to facilitate future development of more potent and pharmacokinetically stable small-molecule c-KIT inhibitors.

## 1. Introduction

The majority of mesenchymal neoplasms of the digestive system are gastrointestinal stromal tumors (GISTs), which develop from interstitial Cajal cells [1,2]. GISTs typically manifest as a sharply demarcated submucosal or subserosal mass in the small intestine (25%) and stomach (60%), but can also occur less commonly in the colon, rectum, esophagus, mesentery, and omentum [3,4]. They often have spindled (70%), epithelioid (20%), or mixed (10%) cytomorphology characteristics. GISTs associated with neurofibromatosis type 1 are typically found in the stomach, have a distinctive multilobular or plexiform architecture, and exhibit either an epithelioid or mixed morphology, but almost never a pure spindle-cell morphology [5]. They are also more likely to occur in multiple locations and to have a spindle-shaped morphology. Anemia, indigestion, bleeding, and abdominal pain brought on by stressful situations are some of the typical clinical signs and symptoms of GISTs [6].

**Figure 1:**
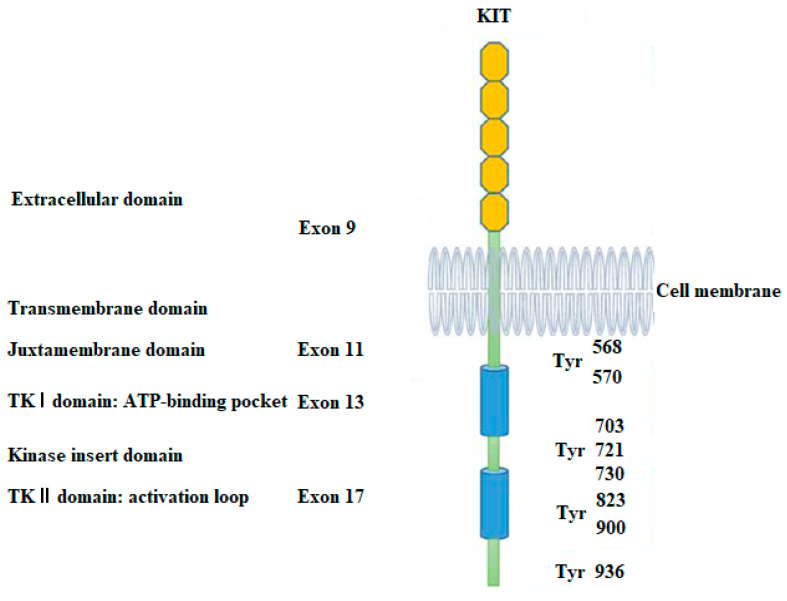
Structure of the KIT receptor. The main mutation sites and phosphorylation sites of KIT in GISTs. Reprinted and modified from Ref. [15].

The KIT receptor tyrosine kinase gene, which codes for c-KIT (also known as mast/stem cell growth factor receptor, SCFR, or CD117), has been found to have primary activating mutations in approximately 90% of GISTs. c-KIT is a type III receptor tyrosine kinase that is present on the surface of hematopoietic stem cells and other cell types [7,8]. When it interacts to its cognate ligand stem cell factor (SCF), it becomes activated. A role in the control of cell survival, proliferation, and differentiation is played by this binding, which leads to the production of receptor dimers, which, in turn, phosphorylate and activate signal transduction molecules in the cell [9,10,11]. Exon 11, which codes for the JM region and destroys its autoinhibitory function, is where c-KIT mutations most frequently occur [12]. This results in continual activation of c-KIT. One of the hotspots of the c-kit gene, codons 550 and 560, is where the majority of these exon 11 mutations occur as deletions and clusters [13]. Other, less frequent mutations in exon 9, which codes for the EC region of c-KIT, include an internal tandem duplication of Ala502-Tyr503, which simulates the conformational change that occurs when c-KIT dimerizes after binding to SCF [13,14]. With a combined frequency of 1–2% across all GISTs, mutations in exons 13 (encoding the ATP-binding region of c-KIT) and 17 (encoding the activation loop of the kinase) are extremely uncommon (Figure 1) [15].

Cancer stemness, also known as the cancer stem cell (CSC) phenotype, is described as a subset of cancer cells that have the capacity to self-renew, specialize into specific progenies, initiate tumor growth, and promote metastasis, recurrence, and therapy resistance [16,17,18]. In several malignancies, stemness has been connected to c-KIT. Research using spheroid cultures obtained from tumor cells from patients with colon cancer grown in serum-free and non-adherent plates—a technique frequently used to study CSCs—have linked c-KIT expression to cancer stemness, and mounting evidence implies a role of c-KIT in colon cancer stemness [19,20]. Recent investigations have shown that more differentiated colon tumor cells release SCF, which affects the proliferation of CSC-like colon tumor cells that express c-KIT [21]. This suggests the possibility of a paracrine mechanism wherein SCF can activate CSC-like cells present in colonospheres. Recent research has also shown that c-KIT increases CSC characteristics in colorectal cancer cells, including the expression of CD44 and other stem cell markers [22,23]. 

**Figure 2:**
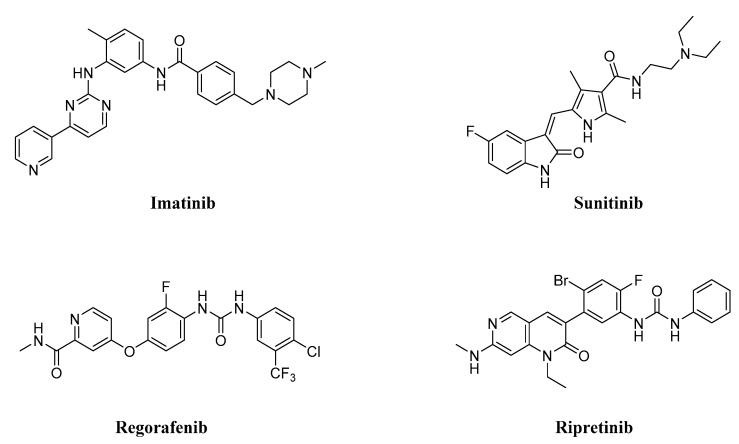
Chemical structures of KIT inhibitors approved for the treatment of GISTs.

Imatinib, a c-KIT inhibitor, has been shown to be effective in treating GISTs, but it has also been linked to primary resistance in some oncogenic mutations, and secondary resistance has frequently emerged [24,25]. Imatinib therapy has a success rate of roughly 70% in the treatment of GISTs with main mutations [26]. After an average of two years, however, acquired resistance is seen in 40–50% of cases [27,28]. Patients with imatinib-resistant GISTs most frequently have the V654A exon 13 resistance mutation [29]. Sunitinib, regorafenib, and ripretinib, which have been approved for the treatment of GISTs, only partially target these resistance mutations and do not improve the overall response rate, as evidenced by their limited therapeutic benefit. Furthermore, these therapies are associated with significant adverse effects (Figure 2) [30,31,32,33]. Thus, there is a critical need for the creation of effective inhibitors for the resistance mutation KIT V654A. Herein, small-molecule c-KIT inhibitors that have been reported to be effective against GISTs are reviewed from a structure–activity relationship (SAR) perspective. It is important to note that only reported c-kit inhibitors with a sufficient number of derivatives to derive a SAR were discussed. Moreover, for each discussed series, the synthetic route as well as the binding pattern (if available) are discussed in detail in an effort to accelerate the discovery of novel c-KIT inhibitors for the treatment of GISTs. The synthesis and Schemes S1–S25 for all the compounds are available in the supplementary information (ref. [34,35,36,37,38,39]).

## 2. c-KIT Inhibitors Targeting GIST

Wang et al. designed and synthesized a novel series of substituted *N*-(4-methyl-3-(piperidin-4-yloxy)phenyl) amide derivatives, and the 27 synthesized compounds were screened for type-II c-KIT inhibition activity for GISTs against the Tel-c-KIT-BaF3, Parental BaF3, K562 cell lines [40]. EL-c-KIT-BaF3 and parental BaF3 cells were employed to monitor the activity, while K562 cells were used to monitor BCR-ABL inhibitory activity. The CF_3_ group substitution at the meta-position of the benzene as R3 was selected as a beginning point for the SAR study. Replacement of the aromatic amine linker of **I** to a flexible 2-carbon aliphatic ether linker at the R2 fragment (**6a** and **6b**) showed a 20–25-fold loss of growth inhibitory activity against TEL-c-KIT-BaF3 cells. After replacement of the aliphatic chain with the piperidine ring (**6c**), the c-KIT activity was improved by 3–4-fold compared with compound **I** (GI_50_ = 0.11 vs. 0.40 μM) and demonstrated good selectivity between TEL-c-KIT-BaF3 and the parental BaF3 cells (GI_50_ ≥ 10 μM). In addition, this compound showed moderate BCR-ABL inhibitory activity (GI_50_ = 2.94 μM). Relocation of the R1 fragment nitrogen position (**6d** and **6e**) led to the complete loss of BCR-ABL inhibitory activity but maintained c-KIT inhibitory activity (GI_50_ = 0.33 and 0.19 μM, respectively) and selectivity over the parental BaF3 cells (GI_50_ ≥ 10 μM). Replacing pyridine with a five-membered heterocyclic group (**6f**) in R1 drastically improved the BCR-ABL activity (GI_50_ = 0.16 μM). Loss of c-KIT activity was observed when the aromatic ring in the R1 fragment was replaced with aliphatic chains, such as ethyl (**6g**) and ethylene (**6h**). The c-KIT inhibitory activity was abolished when the ether linkage was shifted to the three-position from the four-position of the piperidine (**6i** and **6j**) in R2, along with the replacement of the methyl group to a hydrogen atom in R4. Furthermore, a 4–5-fold loss of c-KIT activity (GI_50_ = 0.49 vs. 0.11 μM) was observed when the methyl group in R4 of **6c** was replaced with a hydrogen atom (**6k**). When the methyl group was substituted with an electron-withdrawing group (-Cl), compound **6l** displayed a nine-fold loss of activity against c-KIT (GI_50_ = 0.96 μM), and when it was substituted with an electron-donating group (-Ome), compound **6m** showed complete loss of c-KIT activity (Table 1).

**Figure 3:**
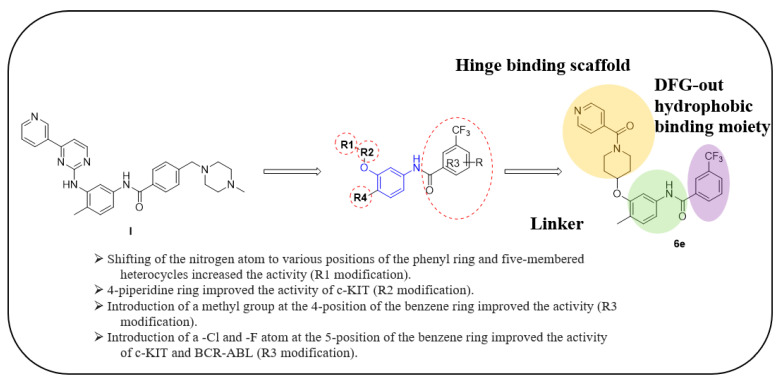
SAR summary and pharmacophore description of substituted *N*-(4-methyl-3-(piperidin-4-yloxy)phenyl) analogs.

These results demonstrate that the piperidine ring in the R2 fragment and the methyl ring in the R4 fragment play important roles in achieving high inhibitory activity. By keeping intact the ether linked piperidine at R2, the introduction of a methyl group at the four-position of the benzene ring (**6n**) led to a six-fold improvement in the c-KIT activity (GI_50_ = 0.031 vs. 0.11 μM) through a loss of selectivity against K562 (GI_50_ = 2.32 μM) and BaF3 cells (GI_50_ = 6.39 μM). Replacement of the R1 fragment with other heterocyclic groups (**6o**, **6p**, and **6q**) and the introduction of a –Cl (**6r**, **6s**) or –F (**6t**) atom at the five-position of the benzene ring in the R3 fragment led to improved BCR-ABL inhibitory activity and c-KIT inhibitory activity, respectively. The pyridine ring in R3 (**6u** and **6v**) resulted in complete loss of activity against c-KIT and BCR-ABL, and the replacement of the CF_3_ group in R3 with a methoxy group (**10a**) led to 15-fold activity loss against c-KIT compared with **6c** (GI_50_ = 1.48 vs. 0.11 μM). Alteration of the benzene ring in R3 to a *tert*-butyl isoxazole moiety (**10b**) led to three-fold activity loss against c-KIT (GI_50_ = 0.34 vs. 0.11 μM), whereas alterations to benzodioxole (**10c**) and quinolone (**10d**) moieties led to a 50-fold loss of c-KIT activity. The introduction of a urea linkage (**13**) between the R3 fragment and the linker moiety in place of an amide increased the BCR-ABL activity and decreased the c-KIT activity (Table 2). Of the tested compounds, **6e** displayed selectivity over ABL kinase and showed potent inhibition against c-KIT kinase. SAR study of this series disclosed that the introduction of a methyl group at the four-position of the benzene ring and piperidine ring in the R2 position was responsible for the c-KIT inhibitory activity. In addition, the terminal pyridine moiety is important to gain selectivity against c-KIT over ABL (Figure 3).

Compound **6e** inhibited c-KIT kinase (IC_50_ = 99 nM) and PDGFRβ (IC_50_ = 120 nM) in the ADP-Glo assay and DDR1 kinase (IC_50_ = 126 nM) and CSF1R (IC_50_ = 133 nM) in the Z’lyte assay. In addition, **6e** showed a strong inhibition in the growth of KIT-dependent GIST cancer cells, such as GIST-T1 (GI_50_ = 0.021 μM) and GIST-882 (GI_50_ = 0.043 μM). Surprisingly, it did not show potent inhibitory activity against the c-KIT-independent GIST cell line GIST48B (GI_50_ ≥ 10 μM), FLT3 kinase activity, and did not exhibit antiproliferative activity against BCR-ABL-driven CML cell lines, such as K562 (GI_50_ ≥ 10 μM), MEG-01 (GI_50_ = 7.43 μM), and KU812 cells (GI_50_ = 6.71 μM). Moreover, **6e** showed a good safety profile over normal Chinese hamster ovary cells, CHO and CHL cells, and leukemic cell lines, such as U937, HL-60, and REC-1 cells. Pharmacokinetics analysis of compound **6e** demonstrated a half-life of 4.11 h and satisfactory bioavailability (F = 36%) when administered orally. In contrast, **6e** exhibited fast clearance (CLz, 4.988 L/h/kg) and a short half-life (T1/2, 0.45 h) when administered intravenously. The antitumor effectiveness of **6e** at 50 and 100 mg/kg doses in a GIST-T1 cell-inoculated xenograft mice model greatly inhibited tumor development.

**Figure 4:**
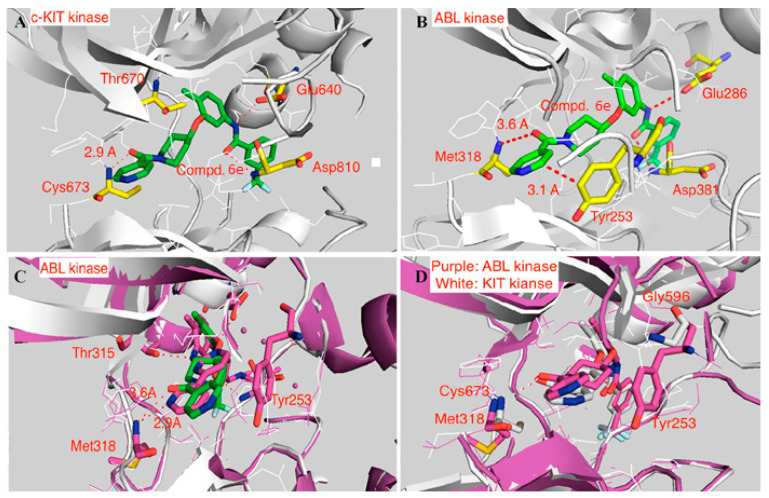
(**A**) docking of **6e** into c-KIT kinase (PDB ID: 1T46) (**B**) docking of **6e** into ABL kinase (PDB ID: 2HYY) (**C**) Superimposition of docking model of **6e** with ABL kinase (PDB ID: 2HYY) (in white) and 1-ABL kinase X-ray crystal structure (in purple). (**D**) Superimposition of **6e** with c-KIT kinase (PDB ID: 1T46) (in white) and docked model of **6e** with ABL kinase (PDB ID: 2HYY) (in purple). Reprinted with permission from Ref. [40].

Molecular docking of the potent derivative **6e** into c-KIT kinase and ABL kinase revealed that **6e** might adopt the DFG-out conformation of c-KIT kinase (PDB ID: 1T46) with a typical type II binding mode, as well as a similar type II binding mode, in the ABL kinase (PDB ID: 2HYY). The amide carbonyl linking the terminal pyridine and piperidine moiety of **6e** forms an O-H-N hydrogen bond with the Cys673 at the hinge-binding region; in contrast, compound **I** uses the terminal pyridine as a hinge binder. The amide moiety in the tail section of **6e** forms two usual hydrogen bonds with Glu640 and Asp810 (Figure 4A). The hydrogen bond established in the hinge-binding region of the ABL kinase (PDB ID: 2HYY) is somewhat longer than that of the c-KIT kinase (3.6 vs. 2.9 Å). Furthermore, the adjacent Tyr253 in the P-loop (3.1 Å to the pyridine moiety) creates a potential steric hindrance that hinders **6e** from binding to the ABL kinase, which was clearly illustrated when superimposing **6e** and **I** in the X-ray crystal structure of 1-ABL kinase. The pyridine moiety in **I** establishes a hydrogen bond with a distance of 2.9 Å in the hinge-binding region, and this aromatic moiety moves further away from Tyr253 than the pyridine moiety in **6e**, probably to prevent potential steric hindrance. Tyr253 was replaced by Gly596 in the c-KIT kinase to accommodate the terminal pyridine (Figure 4).

Li et al. designed and synthesized a series of 6,7-dimethoxy-4-phenoxyquinoline derivatives, and the 30 synthesized compounds were tested for their kinase inhibitory activity against c-KIT wt and c-KIT-T670I using BaF3-TEL-c-KIT, BaF3-TEL-c-KIT-T670I, and parental BaF3 cells [41]. Compound **22a**, which contains a urea group instead of the amide group in compound **I**, displayed enhanced activity for c-KIT wt (GI_50_ = 0.12 μM) and c-KIT-T670I (GI_50_ = 0.071 μM) and maintained a 12-fold selectivity against parental BaF3 cells (GI_50_ = 1.5 μM). However, compound **33**, which had a much larger group (*N*,*N*′-dimethylcyclopropane-1,1-dicarboxamide) in place of the amide group, showed no kinase inhibitory activity (GI_50_ ≥ 10 μM). The R2 moiety was investigated with R1 fixed as urea. With the trifluoromethyl group removed, Compound **22b** completely lost activity against c-KIT wt and c-KIT-T670I (GI_50_ ≥ 10 μM), indicating the importance of the hydrophobic interaction between the CF_3_ group and the hydrophobic environment for binding. In contrast, substituting the phenyl group with a piperidine group (**27**) resulted in a significant loss of activity in BaF3-TEL-c-KIT-T670I cells (GI_50_ ≥ 10 μM) (Table 3). According to these findings, the (trifluoromethyl)benzyl group at position R2 and the urea group at position R1 are favored for better activity. They focused on exploring the R3 position, which is known to enhance the binding affinity and selectivity for type II inhibitors (Table 4). Shifting the propionyl group to position three (**22c**) from position four (**22a**) decreased activity against BaF3-TEL-c-KIT-T670I cells by 13-fold (GI_50_ = 0.92 μM). However, substituting the propionyl group in **22a** with an acetyl group (**22d**) maintained activity against BaF3-TEL-c-KIT (GI_50_ = 0.11 μM) and BaF3-TEL-c-KIT-T670I cells (GI_50_ = 0.046 μM) with improved selectivity against parental BaF3 cells (GI_50_ = 7.1 μM). A ten-fold decreased activity against the c-KIT T670I mutant was observed for larger group substitutions, such as dimethylbutyl (**22e**), glycine (**22f**), and alanine (**22g**).

**Figure 5:**
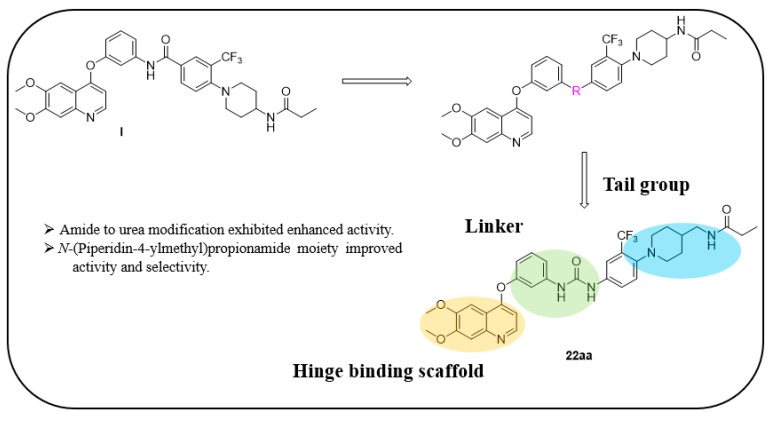
Discovery, SAR summary, and pharmacophore description of quinoline-based urea derivatives as c-KIT wt and c-KIT T670I inhibitors.

However, *N*,*N*-dimethylglycine (**22h**) exhibited improved activity against c-KIT wt (GI_50_ = 0.038 μM) and regained activity against the c-KIT T670I mutant (GI_50_ = 0.053 μM) while maintaining good selectivity toward parental BaF3 cells (GI_50_ = 2.9 μM), compared with **22a**. The addition of cyclohexene (**22i**) and pyridine groups (**22j**) decreased the activity five- to ten-fold, while the addition of tetrahydropyran (**22k**) led to the regained activity against both c-KIT wt (GI_50_ = 0.087 μM) and c-KIT T670I (GI_50_ = 0.083 μM). *N*-Methylpiperidine (**22l**) and *N*-ethylpiperidine (**22m**) showed a similar potency. *N*-Acyl (**22n**), *N*-cyclopropanecarbonyl (**22o**), and Boc (**22p**) led to a two- to three-fold reduction in activity, and 2-methylpiperidine (**22q**) showed a ten-fold decreased activity against c-KIT T670I. Selectivity window to parental BaF3 cells (**22r** and **22s**) or reduced activity against c-KIT T670I (**22t** and **22u**) was observed by increasing the size of the piperidine-derived substituents. However, ethyl-linked morpholine (**22v**) displayed impressive activity against c-KIT wt (GI_50_ = 0.042 μM) and c-KIT T670I (GI_50_ = 0.059 μM), with good selectivity for parental BaF3 cells (GI_50_ = 3.6 μM). Switching the amide moiety to sulfonamide derivatives (**22w**–**22y**) with different aliphatic chains did not improve antiproliferative efficacy against c-KIT T670I mutant. However, introducing the cyclopropyl group (**22z**) regained the activity against BaF3-TEL-c-KIT-T670I cells (GI_50_ = 0.063 μM). *N*-(piperidin-4-ylmethyl)propionamide (**22aa**) improved activity against c-KIT wt (GI_50_ = 0.057 μM), maintained activity against c-KIT T670I, and improved selectivity to parental BaF3 cells (GI_50_ > 10 μM), whereas further enlargement (**22ab**) showed significantly reduced activity, compared with **22aa**. A SAR study of this series revealed that by switching the amide linkage to the urea linkage and increasing the size of the substituent that is coupled to the urea, moiety improved the activity and selectivity against the c-KIT and c-KIT T670I mutant (Figure 5).

Compound **22aa** exhibited potent antiproliferative activity against both c-KIT wt-expressing GIST cancer cell line GIST-T1 (GI50 = 0.004 μM) and the c-KIT T670I-expressing GIST cancer cell line GIST-5R (GI50 = 0.026 μM). The pharmacokinetics of **22aa** demonstrated a half-life of 2.04 h and clearance of 2.76 L/h/kg when administered via intravenous injection. However, oral administration of **22aa** resulted in negligible absorption, which precluded its use via the oral route in the animal model. The antitumor effect of **22aa** at 25, 50 and 100 mg/kg dosages in a BaF3-TEL-c-KIT-T670I cell-inoculated xenograft mouse model was evaluated. Over a period of 10 days of continuous treatment, the growth of BaF3-TEL-c-KIT-T670I tumors was found to be dose-dependently inhibited by compound **22aa**. At a dosage of 100 mg/kg, the compound exhibited a tumor growth inhibition of 47.7%.

**Figure 6:**
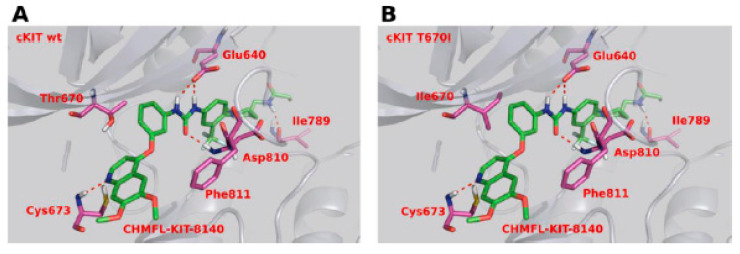
(**A**) Molecular modeling of compound **22aa** with c-KIT wt (PDB code 1T46); (**B**) Molecular modeling of compound **22aa** with the c-KIT T670I homology model (generated based on PDB code 1T46). Reprinted with permission from Ref. [41].

Molecular modeling showed that compound **22aa** adopted a type II binding mode. In c-KIT, the nitrogen atom of the quinoline moiety established a hydrogen bond with Cys673 of the c-KIT domain at the hinge-binding region, and the urea moiety NHs interacted with Glu640 via a hydrogen bond formation. The hydrophobic pocket was occupied by the piperidine moiety and amide NH to form an additional hydrogen bond with Ile789 (Figure 6A). A similar type II binding mechanism was adopted by compound **22aa** in the c-KIT T670I mutant homology model. The O-bridged phenyl moiety in **22aa** was oriented at an angle that allowed room for the large residue isoleucine due to the three hydrogen bonds created by the urea moiety (Figure 6B).

In continuation to their previously developed c-KIT inhibitor **CHMFL-KIT-8140**, Wu et al. designed and synthesized a series of substituted *N*-(4-((6,7-dimethoxyquinolin-4-yl)oxy)phenyl)acetamide derivatives [42]. The 35 synthesized compounds were screened for their inhibitory activity against IL-3-independent BaF3 cells expressing c-KIT wt (BaF3-tel-c-KIT) and c-KIT T670I (BaF3-tel-c-KIT-T670I). The inhibitory activity of **CHMFL-KIT-8140** was significantly reduced by replacing the urea fragment (L2) with acetamide and the R1 tail moiety with a phenyl ring (**35a**) or pyridine (**35b**). Modification of L2 in **35a** to cyclopropanecarboxamide (**36**) did not increase the potency. Nevertheless, introducing a CF3 group at the para-position of R1 (**35c**) significantly increased the activity against c-KIT wt and c-KIT T670I with strong selectivity against parental BaF3 cells (GI_50_ = 2.16 μM) and GI_50_ values of 0.022 μM and 0.011 μM, respectively. Similar efficacy against c-KIT wt and c-KIT T670I was demonstrated by an analogous molecule with an m-CF3 substituent (**35d**) (GI_50_ = 0.020 and 0.001 μM, respectively). Significant activity loss occurred when the R1 substituent was changed from m-CF_3_ to m-F (**35e**) or m-OMe (**35f**). The p-F substitution added to R1 in **35d** (**35g**) resulted in a 13-fold decrease in activity against c-KIT T670I. Cl substitution (**35h**), however, increased the selectivity for parental BaF3 cells (GI_50_ = 5.97 μM) and efficacy against c-KIT wt and c-KIT T670I. The activity against c-KIT wt and c-KIT T670I was slightly decreased by installing a larger substituent, such as a methyl group, at R1 (**35i**). The effects of various substituents at the meta- and para-positions of the benzene ring (**35k**–**m**) were further investigated, and this led to a considerable loss of activity against c-KIT wt. In addition, activity loss was observed when a F or CF_3_ group was installed to the benzene ring ortho-position (**35n**–**p**). Larger groups at R1, such as benzodioxole (**35q**), naphthyl (**35r**), and aliphatic rings such as cyclohexyl (**35s**) and *N*-methyl piperazine (**35t**), led to a loss of activity. When the amide of L2 in **35c** (**43**) and **35d** (**44**) was changed to a reversed amide, activity loss occurred (Table 5).

**Figure 7:**
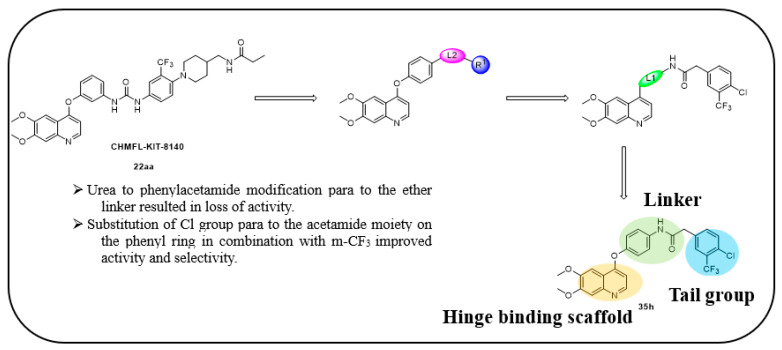
Discovery, SAR summary, and pharmacophore description of compound **35h** from **22aa** to improve the pharmacokinetic profile.

To further explore the SAR of the compound series, they focused on the L1 moiety while fixing the L2 and R1 moieties as phenoxyacetamide and 4-chloro-3-(trifluoromethyl)phenyl, respectively (Table 6). Replacement of the oxygen atom in the L1 linker of compound **35h** with nitrogen (**48**), *N*-methyl (**50**), or sulfur (**52**) resulted in a significant loss of activity against both c-KIT wt and c-KIT T670I. The effects of various substituents on the benzene ring of the L1 moiety was investigated while the phenoxy group was kept intact. The addition of an F atom to the acetamide ortho-position (**39**) improved the activity against both c-KIT wt and c-KIT T670I (GI_50_ = 0.049 and 0.018 μM, respectively). Replacement of the F atom with a chlorine atom (**35a**) or a methyl group (**40a**) displayed similar activity compared to **39**. However, shifting the F atom from the ortho- to the meta-position (**40**) decreased the activity against c-KIT wt (GI_50_ = 0.116 μM). Substituting the meta-position with various groups, such as chloro (**40b**), methyl (**40c**), methoxy (**40d**), trifluoromethyl (**40e**), and nitrile (**40f**), all resulted in a loss of activity compared with compound **35h**. SAR study of this series disclosed that changing the urea linkage to a phenylacetamide linker and substituting the tail phenyl ring with a chloro group in combination with the trifluoromethyl group para to the acetamide linker improved the activity and selectivity (Figure 7).

Compound **35h** showed a strong growth inhibition in KIT-dependent GIST cancer cells, such as GIST-T1 and GIST-882 (GI50 = 0.013 and 0.006 μM) and maintained a selectivity window against the GIST-48B cancer cell line (GI50 = 1.37 μM). When administered orally, the pharmacokinetics of compound **35h** exhibited half-lives of 4.5 h, 6.4 h, and 19.4 h in mice, rats and dogs, respectively. Compound **35h** has an acceptable bioavailability in mice (F = 43%), rats (F = 50%), and dogs (F = 81%). The antitumor effect of **35h** at dosages of 20, 40, 80 and 100 mg/kg in a BaF3-tel-c-KIT-T670I cell-inoculated xenograft mouse model was evaluated. Over an 11-day course of treatment, compound **35h** showed a dose-dependent inhibition of BaF3-tel-c-KIT-T670I tumor progression with almost 100% tumor growth inhibition at a dosage of 100 mg/kg/day.

**Figure 8:**
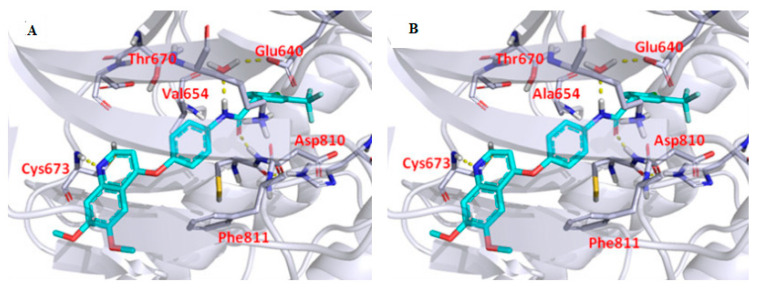
Molecular docking analysis of **35h** (**A**) **35h** with the c-KIT wt (PDB code: 6GQK); (**B**) **35h** with the c-KIT V654A homology model (PDB code: 6GQK); Reprinted with permission from Ref. [42].

The docking results revealed that the potent derivative **35h** adopted a type II binding mode in the c-KIT wt (Figure 8A) and c-KIT T670I mutant. At the hinge-binding region, the nitrogen atom of the quinoline moiety forms a hydrogen bond with Cys673. Two more hydrogen bonds were established by the amide group with Asp810 and Glu640, and the tail phenyl group fit into the hydrophobic pocket in the c-KIT wt model (Figure 8B). 

Liu et al. designed and synthesized structurally modified derivatives of the FDA-approved drug axitinib and tested them for their inhibitory activity [43]. The inhibitory activity of the 29 synthesized compounds was evaluated against IL-3-independent BaF3 cells (GI_50_) expressing c-KIT wt (BaF3-tel-c-KIT) and c-KIT T670I (BaF3-tel-c-KIT-T670I). When the phenyl thiol ether linkage was replaced with a malonamide linkage (**57a**), it showed an improved potency against c-KIT wt and c-KIT T670I (GI_50_ = 0.025 and 0.002 μM, respectively). **57a** also displayed selectivity against parental BaF3 cells (GI_50_ = 7.4 μM), which indicates effective antiproliferative inhibition. Shorter linkers, such as amide **58a**, ethyleneamide **58b**, and urea **59**, showed a decreased activity against c-KIT T670I. Increasing the linker size (**57b**) decreased the activity against both c-KIT wt and c-KIT T670I (Table 7). 

Removal of vinylpyridine in **57a** with a hydrogen atom (**67a**) or methyl group (**67b**) led to a substantial loss of activity. However, 1-methyl-1*H*-pyrazole, pyridine, and 2-methylpyridine (**67c**–**e**) displayed potent inhibitory activity against c-KIT T670I (GI_50_ = 0.023, 0.045, and 0.059 μM, respectively) and had 7- to 15-fold increased selectivity over c-KIT, indicating the importance of the aromatic head moiety for c-KIT kinase binding. Altering the pyridine in **67d** to 5-(methylcarbamoyl)pyridin (**67f**), fluorophenyl (**67g**), or 3-carbamoylphenyl (**67h**) led to a loss of activity against c-KIT wt and c-KIT T670I. *N*-Methyl formamyl (**67i**) showed a six-fold selectivity over c-KIT wt and good activity against c-KIT T670I (GI_50_ = 0.057 μM). Larger groups, such as 4-*N*-methyl piperazine (**67j**) and *N*-methyl piperazinyl methylene (**67k**), showed more potent activity against c-KIT T670I and c-KIT wt, but also exhibited inhibitory activity against parental BaF3 cells (Table 8). 

According to these findings, vinylpyridine in **57a** was preferred for its increased efficacy against c-KIT T670I and for its higher selectivity for c-KIT wt. The impact of the R2 tail moiety was tested while keeping the head and linker the same (Table 9). Compound **57c**, which has a simple phenyl group at R2, displayed a 19-fold selectivity over c-KIT wt, but showed a reduced activity against c-KIT T670I (GI_50_ = 0.117 μM). Meta-halogen-substituted phenyl groups (**57d**–**f**) were tested at R2 to increase the hydrophobicity and *m*-fluoro (**57d**), showing the best selectivity over c-KIT wt (26–fold) and highest potency against c-KIT T670I (GI_50_ = 0.044 μM). Larger substituent groups, such as *m*-methyl (**57g**), *m*-methoxy (**57h**), *m*-*N*,*N*-dimethyl (**57i**), and *m*-trifluoromethyl (**57j**), showed a reduced activity against c-KIT T670I and poorer selectivity for c-KIT wt. The *m*-*N*-methyl piperazinyl group (**57k**) caused significant activity loss. Fluoro-containing groups, such as *o*-fluoro (**57l**), *p*-fluoro (**57m**), and various multifluoro substituents (**57n**–**p**), resulted in loss a of activity against c-KIT T670I, compared with compound **57d**. 

**Figure 9:**
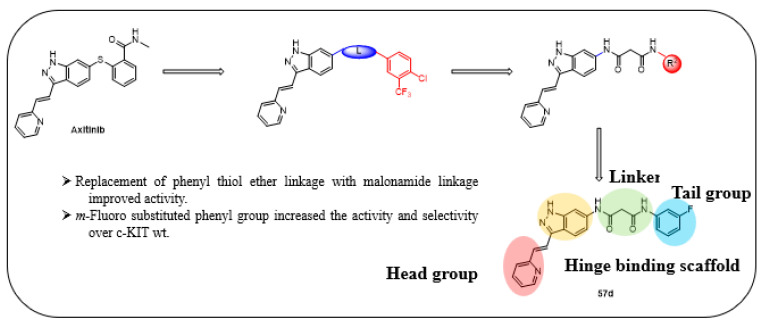
Design, optimization strategy, SAR summary, and pharmacophore description for the discovery of compound **57d**.

A SAR study of this series revealed that the replacement of the phenyl thioether linker with a malonamide moiety and by substituting the tail phenyl ring with meta halogen groups improved the activity and selectivity against c-KIT T670I over c-KIT wt (Figure 9).

The pharmacokinetics of active compound **57d** in a rat model showed 27.5% bioavailability and a half-life of 4.9 h at a dosage of 10 mg/kg when administered orally. In mice, it exhibited a 16.4% bioavailability with a moderate half-life of 1.6 h. The compound had a clearance rate of 12.4 L/h/kg and 6.8 L/h/kg in rats and mice, respectively. The antitumor efficacy of **57d** at a dose of 100 mg/kg in a GIST-5R cell-inoculated xenograft model displayed 83% tumor growth inhibition.

**Figure 10:**
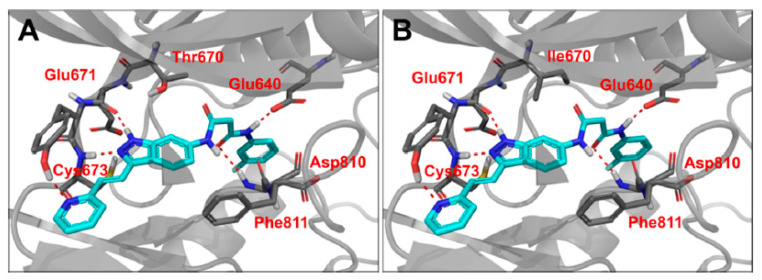
(**A**) Binding mode of **57d** with c-KIT wt (PDB code: 1T46). (**B**) Binding mode of **57d** with c-KIT T670I (PDB code: 1T46). Reprinted with permission from Ref. [43].

A docking study of the potent compound **57d** revealed that the compound adopted a canonical type II binding mode in the c-KIT wt and c-KIT T670I mutant. In the c-KIT wt model, the indazol nitrogen atoms established two hydrogen bonds with Cys673 and Glu671 in the hinge-binding region. Two additional hydrogen bonds formed between the amide moiety and Glu640 and Asp810 at the DFG motif. Furthermore, the hydrophobic pocket was occupied by the tail part. The modeling study was unable to explain the selectivity of compound **57d** against c-KIT T670I over c-KIT wt (Figure 10).

Kaitsiotou et al. designed and synthesized trisubstituted 3-ethynyl-*N*-(4-((4-methylpiperazin-1-yl)methyl)phenyl)benzamide derivatives and screened them against various c-KIT mutants, such as V654A, T670I, and D816H along with wild-type KIT [44]. The design of compounds was started by maintaining the potency of ponatinib and modifying the substitutions in the R1–R4 regions, while the alkyne linker, benzoic acid moiety, and *N*-methylpiperazine moiety of ponatinib were all kept intact throughout the SAR optimization. 

The 24 synthesized derivatives were tested against various c-KIT mutants, as mentioned above. Similar to the potent precursor of ponatinib (**70a**, 6.8 nM), the alkyne precursor (**71a**) displayed a loss of activity (231 nM) against the wild-type KIT. Unsubstituted pyridine analogues (**71b**, 3.5 nM) and (**71e**, 2.1 nM) exhibited similar activity against the wild-type KIT and other KIT mutants. Substitution of an electron-donating group, such as a benzyloxy group at the five-position on the pyridine ring (**71c**), decreased the potency compared with **71b**, whereas methoxy group substitution at the three-position (**71d**) led to a retained potency. Replacement of the pyridine heterocycle with 2-aminopyrimidine (**71g**) increased the potency against the wild-type KIT and the tested mutants (2–247 nM), whereas 3-aminopyridazine (**71f**) retained similar potency against the wild-type KIT but lost activity against all KIT mutants. Substitution of the amine group of 2-aminopyrimidine with an aliphatic chain (**71h**) exhibited an IC_50_ value of 3.3 nM, but lost activity in all KIT mutants compared with **71g**. Further replacements of pyridine, such as 2-aminopyridin-5-yl (**71l**), 2-amino-3-methylpyridin-5-yl (**71j**), and isoquinolin-1-amine (**71m**), resulted in a loss of potency against the D816H and T670I mutant forms. These results suggest that the 2-aminopyrimidine moiety is crucial for potency. Replacing the trifluoromethyl group from the most active derivatives **71a**–**lb**, with a hydrogen atom (**71n**–**q**) and F atom (**71r**–**s**), resulted in a significant loss of inhibitory activity against KIT mutants. These results indicate that the trifluoromethyl group was necessary for inhibitory activity against KIT mutant forms. Shifting the methyl group from the four-position of **71g** to the two-position on the phenyl (**71t**), resulted in a loss of activity against V654A and T670I mutants (Table 10).

**Figure 11:**
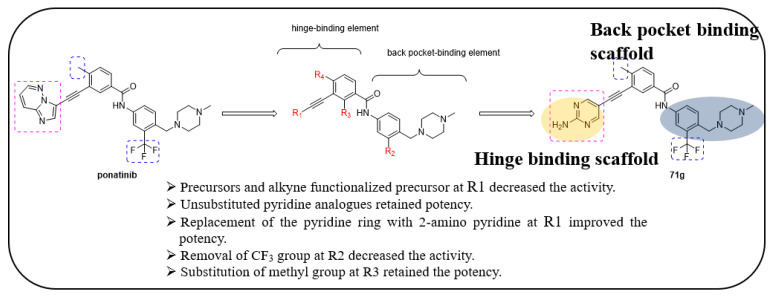
Design strategy, SAR summary, and pharmacophore description of ponatinib structure-modified derivatives.

These derivatives were further screened for their inhibitory activity against GIST-T1, GIST-T1-T670I, GIST430-V654A, and GIST-T1-D816E cells. The results revealed that compounds **71b**, **71d**–**j**, and **71l**–**m** exhibited excellent activity against imatinib-resistant GIST-T1-T670I cells. Compounds **71g**, **71j**, and **71l** demonstrated potent antiproliferative activity (GI_50_ = 141 nM, 474 nM, and 221 nM, respectively) against GIST-T1-D816E cells. In addition, it exhibited improved inhibitory potency against GIST430-V654A cells compared with ponatinib (51 vs. 149 nM). The precursor compounds **70a**–**d** and **71a** did not reduce the cell viability in all tested GIST cell lines. The tested compounds did not exhibit inhibitory activity against KIT-negative GIST-48B cell lines. Compounds **71n**–**s** demonstrated weak inhibitory activity over all KIT-positive and KIT-negative cells. Interestingly, compound **71t** retained an inhibitory activity against GIST430-V654A (308 nM) and GIST-T1-D816E cells (381 nM) and showed a decreased activity against KIT-negative GIST cells. A SAR analysis of this series revealed that the trifluoromethyl group at the R2 position was an important structural feature for activity. Additionally, the replacement of the imidazo[1,2-*b*]pyridazine heterocycle with 2-aminopyrimidine significantly improved the activity against all tested KIT mutants, and also against the wild type c-KIT. In addition, the repositioning the methyl group of the phenylcarboxamide moiety resulted in a significant loss of activity against the secondary mutant V654A and T670I mutant (Figure 11).

Western blot analysis demonstrated that **71g** was the most effective compound for inhibiting autophosphorylation in GIST430-V654A and GIST-T1-D816E cells. A pharmacokinetics study of the potent compound **71g** displayed satisfactory clearance in human liver microsomes (CL_int_, 1 μL min^−1^ mg^−1^), 91% human plasma stability, 98.4% to 99.6% plasma protein binding, and good permeability in Caco-2 cells. These data are in the same range as ponatinib and the FDA-approved KIT inhibitors (Table 11).

**Figure 12:**
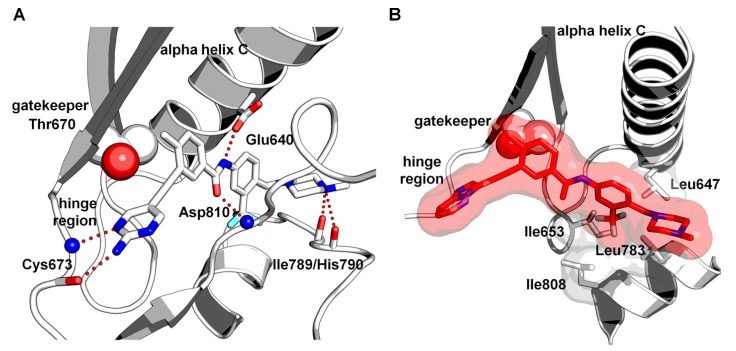
(**A**) Docking of **71g** in complex with wild-type KIT. (**B**) Close-up view of the back pocket. Reprinted with permission from Ref. [44].

The molecular docking of potent compound **71g** and SAR analysis demonstrated that the main hinge binder was a pyrimidine heterocycle. Compound **71g** established two hydrogen bond interactions with Cys673 and Glu640. The amine group of the pyrimidine moiety formed an additional hydrogen bond to the hinge region of the kinases. The trifluoromethyl group was anchored in the back subpocket. The nitrogen atom of the *N*-methylpiperazine moiety displayed H-bond interactions with the carbonyl groups of Ile789/His790 residues (Figure 12).

Kettle et al. designed and synthesized a potent derivative and tested it against the KIT mutant Ba/F3 and PDGFR cell lines to treat GISTs [45]. Screening of previously developed quinazoline-based compounds as PDGFR and VEGFR inhibitors against KIT mutant Ba/F3 cell lines, along with the KDR cell line, resulted in the identification of the lead compound **AZD2932** [46], which exhibited excellent inhibitory activity in this panel along with the KDR cell line. A selective PDGFR inhibitor, compound **I**-**a** [47], which has a central phenoxy ring, displayed less inhibitory activity against KIT mutants and the KDR cell line but showed activity against the parental cell line. Compound **I**-**b**, which has an amine linker instead of a phenoxy linker (**I**-**a**), showed a decreased potency against all the tested cell lines. Methoxy substitution at the meta position on the phenoxy ring (**I**-**c**) led to retained potency, as well as improved KDR selectivity. Replacement of the phenyl ring with meta-pyridine (**I**-**d**) displayed a high selectivity against KDR and an excellent potency against KIT-mutant Ba/F3 cell lines. Quinazoline to quinoline modification (**I**-**e**) led to a retained potency against KIT mutants, but a decreased selectivity against KDR. The combination of quinoline with a meta-methoxyphenol linker (**I**-**f**) resulted in nanomolar activity in all tested cell lines. Compound **II** and its reverse amide (**II**-**a**) (ref. [16,26]) from the PDGFR program demonstrated similar activity against V654A and D816H, but the reverse amide showed a decreased activity against T6701 and improved selectivity against KDR. Methoxy substitution on the central ring (**II**-**b**) did not improve the activity. Quinoline analogues (**III** and **III**-**a**) showed a significant loss of activity in KIT mutants (Table 12; Figure 1).

**Figure 13:**
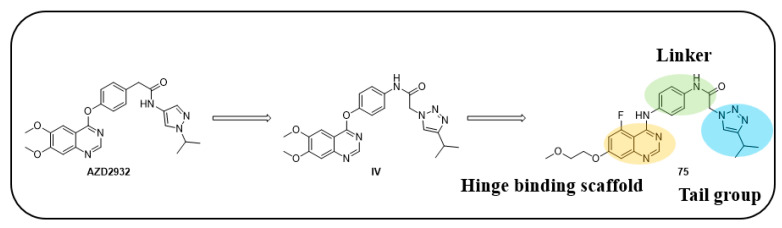
Schematic design, SAR summary, and pharmacophore description for the discovery of compound **75**.

By screening various heterocyclic portions for the modification of isopropyl pyrazole, the N-linked triazole compound (**IV**) was found to exhibit potent inhibition. When the methoxy group was relocated from the six-position to the five-position (**V**), a three-fold loss of activity was observed. Replacement of the ether-linked aromatic ring with the amine-linked aromatic ring (**V**-**a**) led to potent inhibition against KIT mutants along with a 113-fold improved selectivity against KDR. When the five-position methoxy group was replaced with an F atom (**V**-**b**), the activity against KIT mutants was retained, and the selectivity against KDR was improved to a greater extent, but the compound had a 99.7% protein binding. The addition of a methoxyethoxy group (**75**), rather than a methoxy group, retained KIT mutant inhibitory activity and selectivity over KDR. The lead compound, AZD2932, which was identified from their previous work as a PDGFR and VEGFR inhibitor, was utilized to design a potent c-KIT inhibitor. A SAR study of this series revealed that the incorporation of the fluoro group at the five-position of the quinoxaline core improved the potency. Additionally, modifying the methoxy group with an methoxyethoxy group at the seven-position of the core improved the activity against mutant c-KIT. In addition, replacing the quinoxaline ether linker with an amine linker resulted in the retention of the mutant potency and a significant improvement in selectivity over KDR (Figure 13).

The ADME results of **75** indicate that it has good bioavailability (mouse, rat, dog, human: 4.9, 2.2, 6.8, 3.5%), low clearance (mouse, rat, dog, human: 5, 17, <1, <1 μL/min/10–6 cells) and low hERG activity (IC50 = 33.3 μM). Compound **75** showed excellent growth inhibition in all KIT-mutant Ba/F3 cell lines and PDGFR-driven cell lines relevant to the subsets of GISTs, including the clinically GIST-relevant D842V mutant compared with clinically approved and unapproved KIT inhibitors. The in vivo results of compound **75** in a Ba/F3 KIT-exon 11 del/D816H mouse allograft tumor model revealed that there was a 5% inhibition, and tumor size was not affected. However, at a dosage of 20 mg/kg b.i.d., **75** showed excellent regression of tumor volume (75%), and the data are encouraging when compared with regorafenib (39%) at 100 mg/kg q.d. In a Ba/F3 KIT-exon 11 del/V654A mouse allograft tumor model, **75** displayed strong regression (85%) at a dosage of 20 mg/kg b.i.d., but sunitinib showed similar regression (87%) at a dosage of 80 mg/kg q.d.

**Figure 14:**
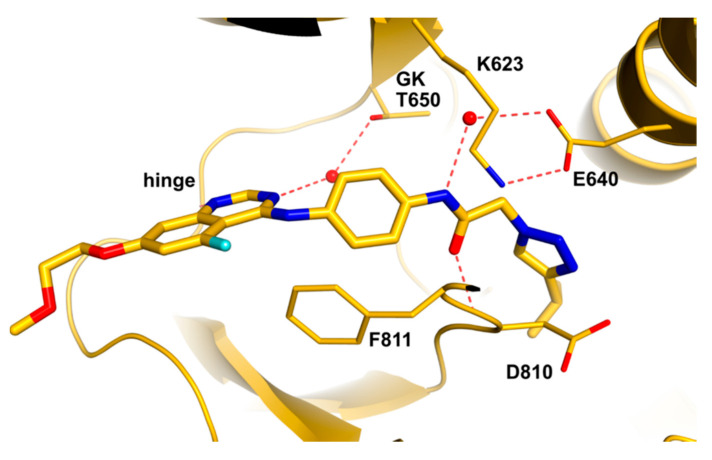
Cocrystal structure of **75** bound to c-KIT (PDB code: 6GQM). Reprinted with permission from Ref. [45].

The cocrystal structure of **75** indicates that the triazolo group binds in the DFG pocket, and there is a water-mediated interaction at the gatekeeper, encouraging selectivity. The C7 side chain was oriented outside of the active site and into the solvent pocket (Figure 14).

**Figure 15:**
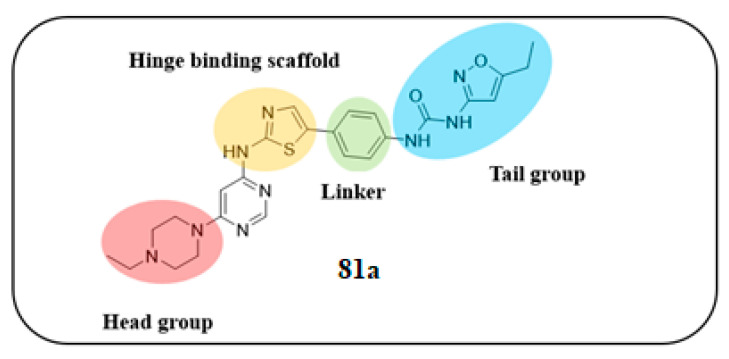
SAR summary and pharmacophore description of **81a**.

Wu et al. designed and synthesized a series of 5-phenyl-thiazol-2-ylamine derivatives, and the 14 synthesized compounds as well as compound **81a** were evaluated for c-KIT kinase activity and GIST-T1 cell proliferation [48]. This demonstrated that 2-methylpyrimidine **81b** had comparable inhibitory effects on both the wild type c-KIT kinase and GIST-T1 cells to compound **81a** (Table 13), while phenyl-substituted urea **81** displayed strong inhibition against c-KIT kinase. Modifications, such as the removal of the 5-ethylisoxazole moiety in **81a** (**81d**) and replacement of the urea component of **81a** with a sulfonamide (**81e**) or an amide (**81f**), led to a reduced cellular potency, but there was no significant impact on c-KIT kinase inhibitory activity. Insertion of a nitrogen atom (**81g**) on the phenyl ring in **81a**, saturation of the phenyl ring (**90**), and replacement of the aromatic ring with an aliphatic chain (**93** and **95**) with the urea component intact did not result in a significant change in inhibitory activity against both c-KIT kinase and GIST-T1 cells. Methyl group substitution at the four-position on the thiazole ring in compound **81h** resulted in a slight decrease in activity against c-KIT kinase and GIST-T1 cells. Shifting the phenylurea tail four-position of the thiazole ring (**84**) led to loss of enzymatic and cellular activity. When the pyrimidine component of **81a** was changed to 2-methyl-1,3,5-triazine (**81i**), it resulted in a six-fold decrease in activity against GIST-T1 cells, revealing the significance of the pyrimidine component in **81a**. Compound **81j**, which has an *N*-(2-hydroxyethyl)piperazine group, demonstrated comparable potency to **81a** in both assays, while compound **81k**, which has a pyrrolidin-3-ylamine group, enhanced enzymatic inhibition against c-KIT but showed lower cellular activity than **81a**. Among the tested compounds, compound **81g** only exhibited slightly improved activity, compared with **81a**, in both enzymatic and cellular assays, but it had a lower synthetic yield (Figure 15).

5-Phenyl-thiazol-2-ylamine pyrimidine template was utilized to design and develop a potent c-KIT inhibitor. A SAR study of this series revealed that substituted phenyl urea was an important structural feature for activity. Additionally, the pyrimidine component was necessary for maintaining the inhibitory activity. In addition, modification of the ethylisoxazole or urea moiety did not affect the c-KIT inhibitory activity (Figure 15).

Notably, compounds **81c** and **81k** exhibited potent inhibition against c-KIT but had unfavorable pharmacokinetic profiles. Thus, compound **81a** was selected for its biological activity and in vivo efficacy. Compound **81a** exhibited potent antiproliferative activity against GIST882, GIST430, and GIST48 (GI_50_ = 3, 1, and 2 nM, respectively).

The pharmacokinetics of compound **81a** in a mouse model demonstrated moderate bioavailability (F = 38%), AUC (2796 ng/mL∙h) and a short half-life (t_1/2_ = 2.8 h) with oral administration. Compound **81a** exhibited a 7.1 L/kg volume of distribution (V_ss_) and a 20.2 mL/min/kg plasma clearance rate (Cl) with intravenous administration. The antitumor efficacy of **81a** at 40 and 25 mg/kg dosages in a GIST430 tumor xenograft model was superior to the FDA-approved sunitinib and showed 59% reduction in tumor size on day 14.

**Figure 16:**
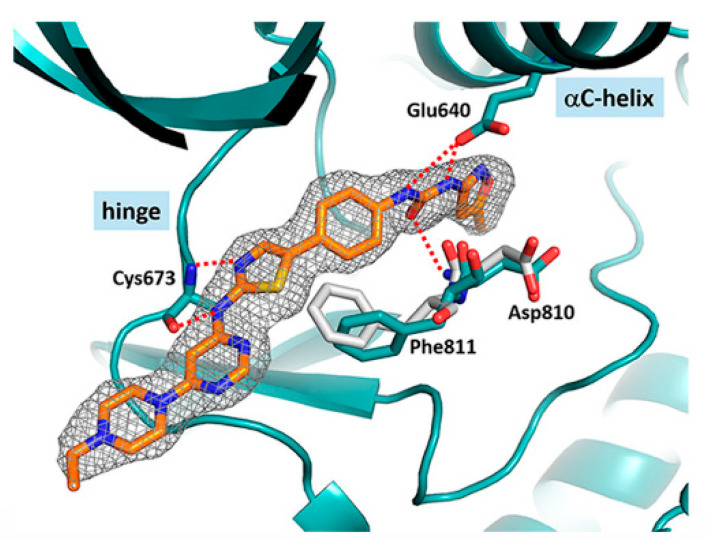
Chemical structure of **81A** and in complex with the unactivated c-KIT kinase domain (PDB 6ITT). Reprinted with permission from Ref. [48].

The molecular interactions of **81a** in the unactivated c-KIT kinase domain displayed type II binding mode (Figure 16). The thiazolylamine formed two hydrogen bonds in the hinge-binding area of c-KIT with the backbone of Cys673. The back pocket of the ATP-binding site was occupied by the tail group, which formed three more H-bonds with the urea group, backbone of Asp810, and side chain of Glu640. The phenyl ring of **81a** formed an aromatic interaction with Phe811, while the ethylpiperazine was oriented toward the solvent region.

**Figure 17:**
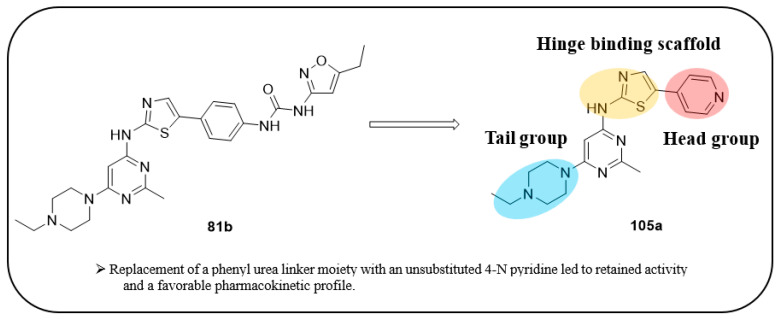
Rational design, SAR summary, and pharmacophore description of compound **105a**.

Lin et al. continued their research from their previous compound **81a** by rational design, and the team synthesized a series of five-aromatic substituted thiazol-2-ylamine pyrimidine derivatives [49]. The 14 synthesized compounds were evaluated against c-KIT and wt-FLT3 kinases GIST-T1 and MOLM-13 cell lines (Table 14). Compound **102** had a higher inhibitory effect against c-KIT (IC_50_ = 24 nM) than **I** (GI_50_ = 35 nM), but lost its antiproliferative activity against MOLM-13 cells. Changing the phenyl ring in **102** to a pyridine ring, led to compound **103**, which had a similar inhibitory potency against c-KIT and FLT3, but decreased cellular potencies (GI_50_ = 42 and 36 nM for GIST-T1 and MOLM-13, respectively) relative to **I**. When the nitrogen atom was shifted to the third position, compounds **104a** (R2 = CH_3_) and **104b** (R2 = H) exhibited similar inhibitory effects against the kinases as compound **I** but were less active in the cellular assays (GI_50_ values of 26–140 nM). Compound **105a** (R2 = CH_3_) had single-digit nanomolar activity against GIST-T1 (GI_50_ = 7.1 nM) and MOLM-13 (GI_50_ = 9.4 nM) cell lines and exhibited similar inhibitory activities against c-KIT and FLT3 compared with **I**. As compared with **105a**, **105b** (R2 = H) displayed no improvement in cellular potency (GI_50_ > 10 nM); however, it exhibited potent inhibitory activities against c-KIT and FLT3 (IC_50_ < 30 nM). Next, the effect of water-solubilizing substituents on the pyrimidine ring four-position was evaluated, and the potency of compounds **105c**–**f** was compared to that of the *N*-ethylpiperazine analog **105a**. Limited water-solubilizing groups were considered in this study. Analogs **105g** and **106** were produced by inserting a methyl group on the pyridine ring of **105a** and changing the pyridine ring of **105a** to a pyrimidine ring, respectively. *N*-(2-fluoroethyl)piperazine, *N*-(2-hydroxyethyl)piperazine, *N*,*N*-dimethylpiperidin-4-amine, and 4-(2-hydroxyethyl)morpholine derivatives (**105c**, **105d**, **105e**, and **105f**, respectively) did not affect the activity against the kinases c-KIT and FLT3. The *N*,*N*-dimethylpiperidin-4-amine group in **105e** increased the cellular activities slightly (GIST-T1 GI_50_ = 1.5, MOLM-13 GI_50_ = 3.5 nM), whereas the morpholine group linked by a two-carbon ether in **105f** led to decreased cellular potency (GIST-T1 GI_50_ = 27 nM, MOLM-13 GI_50_ = 42 nM). The study considered only a few water-soluble groups since the impact of some groups on biological activities, in vivo toxicities, and pharmacokinetics had been well-established during the development of 5-phenylthiazol-2-ylamine-based inhibitors. Analogs **105g** and **106**, with a single methyl substituent on the pyridine ring of **105a** and a pyrimidine moiety instead of pyridine, respectively, were produced. The 2-methylpyridine compound **105g** had a two- to four-fold decrease in potency against c-KIT, FLT3, and MOLM-13 cells compared with **105a**, but retained activity (GI_50_ = 7.7 nM) against GIST-T1 cells. Pyrimidine **106** had a moderate inhibitory activity for c-KIT (IC_50_ = 123 nM) and FLT3 (IC_50_ = 163 nM), but a significantly reduced cellular potency (GI_50_ > 100 nM). Optimization of the 5-pyridin-4-yl-thiazol-2-yl series of pyrimidines (compound **105**) was performed by investigating the effects of substitutions on 2-aminothiazole. When the pyridine ring in **104a** was moved from the five- to the four-position on the thiazole ring (**109**), compounds **109** and **111** showed a significant decrease in both enzymatic activities (IC_50_ > 1000 nM) and cellular potencies (GI_50_ > 1000 nM), compared with **104a**. In response to previous studies that demonstrated the ability of 2-aminothiazole to form hydrogen bonds with the hinge region of the ATP pocket, benzamide **111** was produced by replacing the pyrimidine ring with a solubilized *para*-substituted benzamide ring, which is a recognized scaffold for FLT3 kinase inhibitors. Benzamide **111** was found to have submicromolar activities against MOLM-13 cells (GI_50_ = 544 nM) and GIST-T1 (GI_50_ = 647 nM), but showed moderate inhibitory activity against c-KIT/FLT3. Therefore, no further modification of lead **111** was conducted. The pyridine-substituted 2-aminothiazole analogs **105a** and **150c**–**e** exhibited potent dual inhibition of c-KIT and FLT3 with cellular antiproliferative activities of less than 10 nM (Figure 17).

A SAR study of this series suggested that altering the phenyl urea moiety with a pyridine ring, with its four-position to thiazole, led to the identification of the most potent derivative with a favorable pharmacokinetic profile (Figure 17).

The pharmacokinetics, antitumor activities, and toxicities of **105a** and **150c**–**e** in SCID mouse xenografts or normal mice showed that **105a** has a greater impact on GIST430 xenografts than **105c** and **105d**, as well as a lower toxicity and more favorable pharmacokinetic profile than **105c** and **105e**.

The pharmacokinetics of the potent compound **105a** in male Sprague–Dawley rats and ICR mice demonstrated a bioavailability of 36% in rats and 68% in mice, and a moderate half-life (t_1/2_) of 3.9 h in rats and 4.1 h in mice when administered orally. When used via intravenous administration, **105a** displayed high volumes of distribution (V_ss_ = 14.7 L/kg in mice and 10.1 L/kg in rats) and plasma clearances (Cl = 70.7 mL/min/kg in mice and 393.6 mL/min/kg in rats). The antitumor effect of **105a** at 10, 20, and 40 mg/kg dosages in NOD/SCID mice bearing GIST430 tumors showed rapid tumor regression.

**Figure 18:**
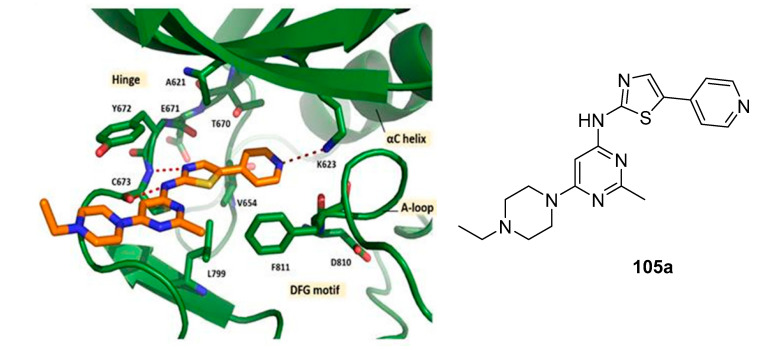
Interactions of **105a** in complex with c-KIT and the chemical structure of **105a**. Reprinted with permission from Ref. [49].

The crystal structure of c-KIT, in complex with compound **105a** (Figure 18), revealed that thiazolylamine formed two hydrogen bonds with Cys673 and established hydrophobic interactions with Leu595, Tyr672, Cys673, and Leu799. The ethylpiperazine group was oriented toward the solvent exposed region. The pyridine moiety formed additional hydrophobic interactions with Leu799 and Ala621.

Lu et al. designed structural modifications to linifanib and synthesized a series of 3-methyl-1*H*-pyrazolo[3,4-*b*]pyridine derivatives [50]. The structural modification of a linifanib (**I**) resulted in the generation of 58 derivatives, which were screened for their inhibitory potency (IC_50_) against PDGFRα, VEGFR2, and FGFR1. Based on their initial optimization (Table 15), compound **122d**, the most potent PDGFRα inhibitor, was selected to explore its activity against other RTKs as it showed potent inhibitory activity against c-KIT with an IC_50_ value of 2.1 nM (Table 16).

This led to the development of a dual inhibitor against c-KIT and PDGFRα, and VEGFR2 kinase was used as a reference to monitor selectivity. To evaluate the impact of various three-position substituents of the pyrazolo[3,4-*b*]pyridine scaffold, the 3-methyl group was replaced with hydroxymethyl (**126a**, **126b**) and trifluoromethyl groups (**122g**, **122h**). The replacement of the 3-methyl group with a hydroxymethyl group (**126a** vs. **122d**) resulted in a two-fold activity reduction in PDGFRα (IC_50_ = 87 vs. 40 nM), while the c-KIT inhibition remained relatively potent (IC_50_ = 2.4 vs. 2.1 nM) (Table 17).

The trifluoromethyl group (**122g**) resulted in complete loss of activity in all kinases (IC_50_ > 50,000 nM). These results revealed that for the dual inhibition of c-KIT and PDGFRα, the optimal pharmacophore was the 3-methyl-pyrazolo[3,4-*b*]pyridine moiety. As a potential template, the urea linker and the 3-methyl-1*H*-pyrazolo[3,4-*b*]pyridine scaffold were used to identify a dual c-KIT/PDGFRα inhibitor. Next, the substitution effect (R2) on the phenyl ring linked to terminal nitrogen of urea was investigated. Shifting the methyl group from the three-position (**122d**) to the two- (**122j**) or four-position (**122k**) resulted in a significant activity loss against c-KIT/PDGFRα. The 3-methyl group was found to be the most effective substituent among several 3-substituents (**112d**–**f**, **122l**–**n**, and **130a**) for inhibiting c-KIT/PDGFRα. Interestingly, when an *N*-methyl piperazinyl group was substituted at the three-position, the inhibitory activity against c-KIT/PDGFRα was moderately reduced. Compounds **130b**–**d** were synthesized with a combination of a 3-methyl group and either an *N*-methyl piperazinyl or a morpholinyl group on the terminal phenyl ring. Compound **130d** showed the most potent inhibitory activity against both c-KIT and PDGFRα kinases (IC_50_ = 2.4 and 7.2 nM, respectively) and maintained selectivity for VEGFR2, suggesting that compound **130d** was a promising candidate for further development as a dual inhibitor. Changing the combination of the *N*-methyl piperazinyl or the morpholinyl or methyl groups (**130e**–**h**) resulted in a significant loss of activity against all three kinases. The introduction of a diethylamino group (**130i** and **130j**) or a dimethylamino group (**130k** and **130l**) on the terminal phenyl ring partially restored this dual activity, but the potency was lower than that achieved with compound **130d**. The replacement of the 3-dimethylamino group with a 3-dimethylaminomethyl group (**130m**) displayed a complete loss of activity against all three kinases. Further screening of the R2 group (**127a**–**y**) was performed to identify more potent compounds (Table 18). While increasing the length of the linear alkyl groups (**127a**–**e**) improved the c-KIT/PDGFRα inhibition activity, replacing the linear alkyl group with a branched (**127f** and **127g**) or cycloalkyl (1**27h**–**k**) group did not result in an improved inhibitory activity compared with the original compound (**122d**). Heteroarylmethyl groups (**127l**–**n**) exhibited better c-KIT/PDGFRα inhibitory activity than the phenylmethyl group (**127o**), but the inhibitory activity was lower than that which was advised with **122d**. By varying the chain length of phenylalkyl groups (**127o**–**s**), the potency against c-KIT/PDGFRα increased in the following order: phenylpropyl (**122q**) > phenylethyl (**127p**) > 2,3-dihydro-indene (**127s**) > phenylbutyl (**127r**). Compound **127q** had better c-KIT inhibitory activity than **122d** and **130d** but showed weaker PDGFRα inhibitory activity than **130d** (IC_50_ = 24 nM vs. 7.2 nM). Heteroaryl groups (**127t**–**v**) reduced the kinase potency by over six-fold when replacing the aryl group (R2). Fused aryl rings (**127w**–**y**) improved c-KIT/PDGFRα inhibition compared with **122d** but not to single-digit nanomolar concentration. The structural optimization generated several novel compounds with an improved potency against the tested kinases. Compound **130d** was particularly notable for its 17-fold improvement in potency, compared with the benchmark imatinib for c-KIT and for its dual potent activity against both c-KIT (IC_50_ = 2.4 nM) and PDGFRα (IC_50_ = 7.2 nM). Other promising compounds include **127e**, **127q**, and **127w**, which also exhibited a high dual-target potency and appropriate selectivity against VEGFR2.

**Figure 19:**
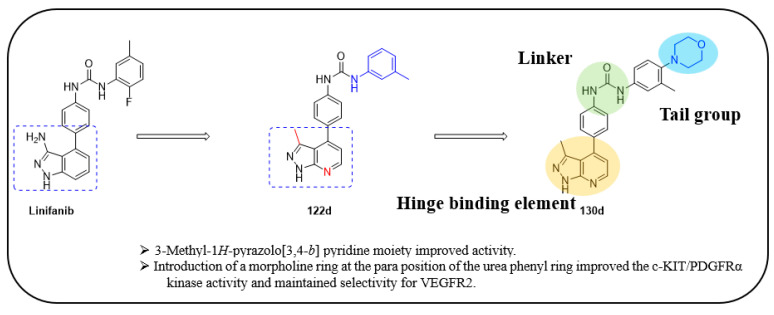
Design strategy, SAR summary, and pharmacophore description of compound **130d**.

A SAR study of this series revealed that the 3-methyl-1*H*-pyrazolo[3,4-*b*]pyridine pharmacophore was an important structural feature for the dual inhibition of c-KIT and PDGFRα. Additionally, it was observed that there was a correlation between the activity and the terminal amines. In addition, the introduction of the morpholine group para to the phenyl urea moiety improved activity by retaining selectivity over VEGFR2 (Figure 19).

The antiproliferation activity of **130d** showed significant improvement against the GIST-T1 (GI50 < 0.003 μM) and GIST-882 cell lines (GI50 < 0.020 μM).The pharmacokinetics of compound **130d** in a rat model displayed a short half-life (t_1/2_) of 0.69 h, a small volume distribution (V_ss_) of 0.97 L/kg, and a moderate plasma clearance (Cl) of 0.95 L/h/kg at a dosage of 2 mg/kg via intravenous administration, while intraperitoneal injection at a dosage of 100 mg/kg improved the plasma exposure and half-life (T1/2 = 10.97 h). In contrast, the lack of absorption at the 10 mg/kg dosage during oral administration prevented **130d** from being utilized for oral administration in the animal model. The in vivo efficacy of **130d** at a dosage of 100 mg/kg in a BaF3-TEL-c-KIT-T670I cell-inoculated xenograft mouse model displayed 41.9% tumor growth inhibition and a good safety profile.

Andreas et al. designed and synthesized 3-(pyrimidin-4-yl)imidazo[1,2-a]pyridine derivatives as selective c-KIT inhibitors for the treatment of GISTs [51]. A high-throughput screening of derivatives supplied by the European Lead Factory resulted in the identification of an imidazopyridine derivative as a hit against KIT (V654A). The hit compound displayed high selectivity against KIT autophosphorylation at Y703 in a GIST430 cancer cell line over 28 kinases. Except for its microsomal stability, the hit compound had good solubility and permeability. The pyrrolidine and benzylic positions were the reason for the high metabolism. At first, hit-to-lead optimization was performed, and the SAR exploration was mainly concentrated on improving cellular potency and metabolic stability. The introduction of 4,6-substituted pyrimidine (**III**) in place of a three-position substitution on the pyridine ring improved the cellular potency, and it was identified as an ideal replacement. Linker region modification (**II** and **IV**) resulted in a three-fold decreased potency. When the pyrrolidine substituted pyridine moiety was replaced with a fused ring system and an aliphatic moiety (**V** and **VI**), there was a complete loss of potency, but the phenyl ring (**VII**) showed a slight increase in cellular potency (Table 19).

Modification of the pyrrolidine moiety with a phenyl ring (**VIII**) led to a further increase in both cellular potency and metabolic stability. Substitution at the six-position of imidazopyridine with electronic-donating groups for ether (**IX**) resulted in increased cellular potency. Increasing the length of the ether linker along with methyl ether substitution in the tail part further increased the cellular potency (**X**). Basic substitutions at the tail part of the extended ether linker increased the water solubility by maintaining the potency (**XI**). Bipheylmethanamine in the benzylic part, in combination with 6-methoxy substitution and 4,6-pyrimidine in the central core, resulted in the identification of compound (**XII**), which had an improved cellular potency. When the terminal part of the phenyl ring was replaced with a five-membered heterocycle (**XIII**), the metabolic stability was retained, and the cellular potency was further improved. The extended ether linker (**135a**) maintained metabolic stability and cellular potency (Table 20). Based on the SAR results, **135a** was identified as a lead compound.

**Figure 20:**
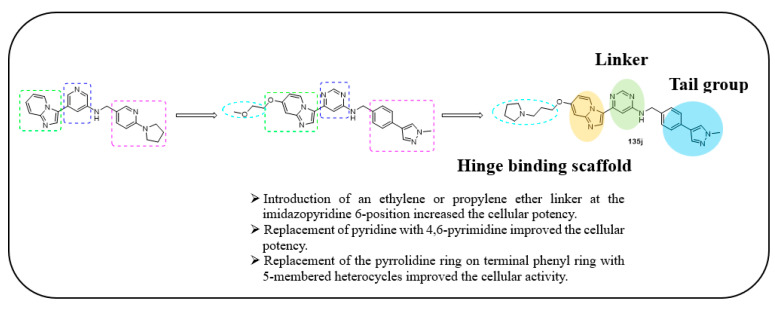
Design strategy, SAR summary, and pharmacophore description of compound **135j**.

The lead optimization of **135a** was focused on improving the solubility, permeability, and hERG inhibition. Terminal pyrazole ring replacement with additional five-membered heterocycles, such as triazole (**136a** and **136b**) and oxazole (**137**), led to a retained cellular potency. The introduction of polar groups at the tail part of the extended ether linker (**135b** and **135c**) reduced the permeability and increased the efflux; however, for **135d** and **135e**, the hERG activity was improved. All the derivatives displayed superior cell potency and kinase selectivity (Figure 20) (Table 21).

The HTS screening of the European Lead Factory derivatives resulted in the identification of an imidazopyridine derivative as a hit against KIT. A SAR study of this series revealed that the 4,6-pyrimide moiety was an important structural feature for cellular potency. In addition, the introduction of an ethylene or propylene ether linker at the six-position of the imidazopyridine core and the replacement of the pyrrolidine ring on the terminal phenyl ring with a N-methyl pyrazole improved the cellular potency. Furthermore, it was observed that the pyrrolidin-1-yl propylene ether linker resulted in improved solubility and strong hERG inhibition (Figure 20).

Across all preclinical species, the pharmacokinetics of **135j** demonstrated great metabolic stability, a good half-life, and a high volume of distribution. Compound **135j** displayed excellent cellular potency (IC_50_ = 4 ± 1 nM) against the imatinib-sensitive GIST430 cell line. In addition, it exhibited good cellular potency in the imatinib-resistant cell line GIST430/654 and the AML cell line Kasumi-1 (IC_50_ = 48 ± 21 and 4 ± 1 nM, respectively). The in vivo results on GIST430/654 xenografts indicated that **132j** strongly inhibited tumor growth. The compound was further evaluated in in vivo studies in dogs and guinea pigs, which revealed its long half-life and lack of accumulation. The compound was nominated for a phase 1 clinical study in humans with metastatic and surgically unresectable GISTs.

**Figure 21:**
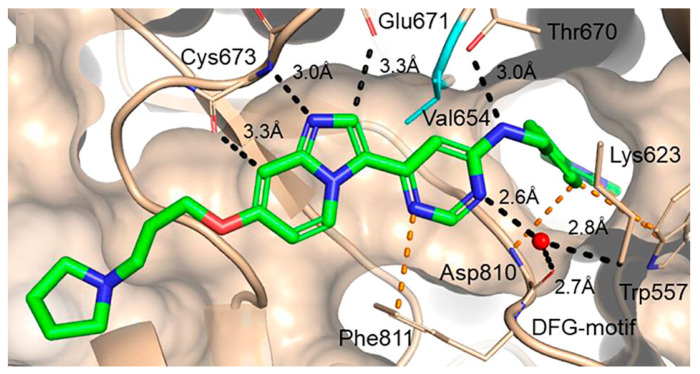
X-ray structure of **135j** (2.1 A resolution, PDB-ID: 7ZW8) in complex with the kinase domain of c-KIT. Reprinted with permission from Ref. [51].

The molecular docking study of **135j** revealed that it could adopt a DFG-out conformation of c-KIT kinase with typical type II binding. The nitrogen of the imidazopyridine established a hydrogen bond with Cys673 in the kinase hinge region. The side chain of the ether linker was oriented toward the solvent exposure region. The benzylic NH formed an interaction with the Thr670 side chain, and the benzylic substituent was deeply embedded via π-interactions with Trp557. One of the pyrimidine nitrogen atoms was seen to engage with the Asp810 backbone carbonyl and the terminal amino group of the Lys623 side chain via the water-mediated process (Figure 21).

**Figure 22:**
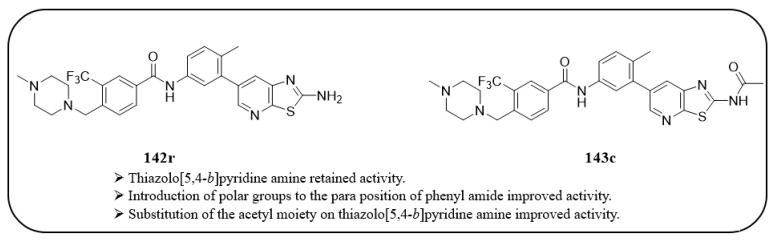
SAR summary and chemical structures of compounds **142r** and **143c**.

Nam et al. designed and reported the synthesis of thiazolo[5,4-*b*]pyridine-based derivatives targeting antiproliferative activities [52].The 31 synthesized compounds were evaluated for their enzymatic inhibitory activity against c-KIT (Table 22). Imatinib and sunitinib were used as standard references with IC_50_ values of 0.27 µM and 0.14 µM, respectively. SAR analysis showed that among the compounds 1**42a**–**j**, the 3-(trifluoromethyl)phenyl group (**142h**) only exhibited moderate enzymatic inhibitory activity (IC_50_ = 9.87 µM). Extension of the amide and introduction of a urea linkage led to a loss of activity. The addition of polar moieties in the para position to the amide group on the 3-(trifluoromethyl)phenyl group resulted in a two- to six-fold improved inhibitory activity, whereas addition of the meta position led to decreased inhibitory activity. By further exploring para substitution with various moieties, the (4-methylpiperazin-1-yl)methyl analog 1**42r** was found to be the most potent derivative (IC_50_ = 0.14 µM). Modification of the amino group of **142r** with cyclohexyl (**143a**) and phenyl amide (**143b**) led to decreased inhibitory activity compared with **142r** (IC_50_ = 0.14 µM vs. 1.51 µM and 0.74 µM, respectively). The acetamide analog (**143c**) displayed little improvement in activity with an IC_50_ value of 0.10 µM. Additional acetamide derivatives (**143f**–**h**) with various para substitutions on the 3-trifluoromethyl phenyl amide moiety were more potent than their precursor compounds (**142p**, **142v**, and **142w**) (Figure 22).

A SAR study of this series revealed that the thiazolo[5,4-*b*]pyridine core was an important structural feature for enzymatic and anti-proliferative activities. Additionally, the introduction of polar groups to the para-position of phenyl amide improved activity. Similarly, the substitution of an acetyl group on thiazolo[5,4-*b*]pyridine amine further improved the activity (Figure 22).

The compounds were further screened for their antiproliferative activity against c-KIT-dependent GIST-T1 and HMC1.2 cancer cell lines (Table 23). The results showed that compounds **142r** (GI_50_ = 0.01 ± 0.00 µM), **142s** (GI_50_ = 0.02 ± 0.00 µM), and **143c** (GI_50_ = 0.01 ± 0.00 µM) exhibited similar antiproliferative activity against GIST-T1 cells compared with sunitinib (GI_50_ = 0.01 ± 0.00), but **142s** (GI_50_ = 1.33 ± 0.43 µM), and **143c** (GI_50_ = 1.52 ± 0.43 µM) displayed a potent antiproliferative activity against HMC1.2 cells (GI_50_ = 1.15 ± 0.96 µM) compared with imatinib (GI_50_ = 27.10 ± 3.36 µM). The enzymatic inhibitory activities revealed that the active compounds showed a five- to eight-fold improved activity against c-KIT V560G/D816V double mutant compared with imatinib (IC50 = 37.93 µM). On the other hand, these compounds displayed similar activity to sunitinib (IC50 = 3.98 µM). In addition, they exhibited similar activity against c-KIT D816V Ba/F3 cells, while having a higher cytotoxicity window for c-KIT D816V Ba/F3 cells compared with parental Ba/F3 cells than sunitinib.

**Figure 23:**
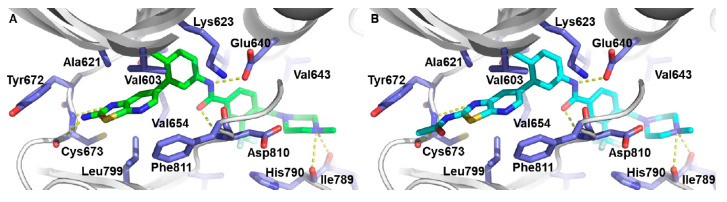
(**A**,**B**) Docking model of compound of **142r** and **143c** on c-KIT (PDB code: 1T46). Reprinted from Ref. [52].

A molecular docking study was performed to elucidate the binding mode of the potent compounds **142r** and **143c**. Both compounds established hydrogen bonding at the hinge region with a Cys673 backbone, as well as with a Glu640/Asp810 and an Ile789/His790 backbone. The thiazolo[5,4-*b*]pyridine fragment was involved in hydrophobic interactions with Leu799, Val603, Ala621, and Val654, and the hydrophobic pocket was occupied by the 3-trifluoromethyl group (Figure 23).

## 3. Conclusions

In conclusion, c-KIT has emerged as a promising target in drug development for the treatment of GISTs, and the emergence of resistance to imatinib has highlighted the need for more potent and selective inhibitors. Various sub-structural modifications have been explored to discover a potent inhibitor, and in this review, we focused on the synthesis and the SAR optimization of various scaffolds for c-KIT inhibitors for GISTs. Several compounds have demonstrated potent c-KIT inhibitory activity and antitumor efficacy, including compounds **6e**, **22aa**, **35h**, **57d**, **71g**, **75**, and **130d**. Compounds **81a** and **105a** have also shown promise as next-generation therapeutic candidates for GISTs. In addition, compound **135j** (IDRX-42) has been designated as a clinical candidate and is undergoing testing in a phase 1 trial. The results of these studies provide insight for medicinal chemists to design new scaffolds targeting c-KIT-driven GISTs.

## Figures and Tables

**Table 1:** SAR exploration of the R1/R2/R4 positions.

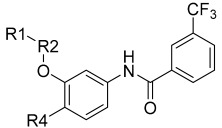						
Compd.	R1	R2	R4	Tel-c-KIT-BaF3 (GI_50_: μM)	Parental BaF3 (GI_50_: μM)	K562 (GI_50_: μM)
**I**	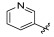	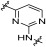	–Me	0.40	>10	0.12
**6a**	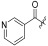	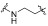	–Me	7.94	>10	>10
**6b**	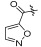	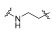	–Me	9.97	>10	>10
**6c**	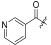	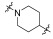	–Me	0.11	>10	2.94
**6d**	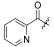	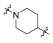	–Me	0.33	>10	>10
**6e**	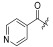	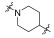	–Me	0.19	>10	>10
**6f**	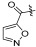	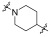	–Me	0.22	>10	0.16
**6g**	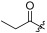	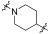	–Me	1.55	>10	>10
**6h**	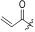	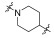	–Me	0.62	>10	8.36
**6i**	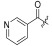	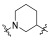	–H	8.07	8.07	>10
**6j**	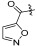	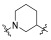	–H	>10	>10	>10
**6k**	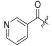	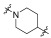	–H	0.49	>10	>10
**6l**	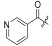	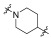	–Cl	0.96	>10	2.8
**6m**	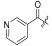	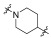	–OMe	>10	>10	>10

**Table 2:** SAR exploration of the R1/R3 positions.

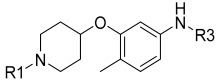					
Compd.	R1	R3	Tel-c-KIT-BaF3 (GI_50_: μM)	Parental BaF3 (GI_50_: μM)	K562 (GI_50_: μM)
**6n**	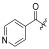	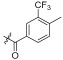	0.031	6.39	2.32
**6o**	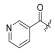	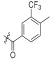	0.083	5.47	5.85
**6p**	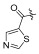	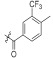	0.10	>10	5.24
**6q**	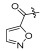	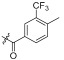	0.065	8.94	1.96
**6r**	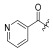	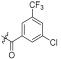	0.16	>10	3.66
**6s**	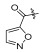	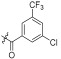	0.15	>10	1.95
**6t**	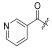	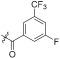	0.15	>10	4.56
**6u**	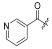	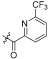	>10	>10	>10
**6v**	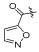	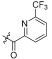	>10	>10	>10
**10a**	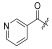	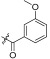	1.48	>10	>10
**10b**	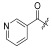	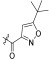	0.34	>10	9.92
**10c**	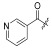	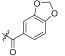	4.8	>10	>10
**10d**	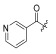	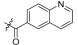	5.55	>10	>10
**13**	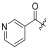	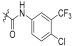	2.6	3.3	0.78

**Table 3:** SAR exploration of the R1/R2 positions.

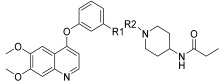					
Compd.	R1	R2	BaF3-TEL-c-KIT (GI_50_: μM)	BaF3-TEL-c-KIT-T670I (GI_50_: μM)	BaF3 (GI_50_: μM)
**I**	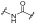	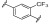	0.4 ± 0.011	2.7 ± 0.058	>10
**22a**	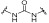	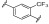	0.12 ± 0.016	0.071 ± 0.0011	1.5 ± 0.17
**33**	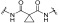	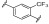	>10	>10	>10
**22b**	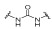	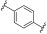	>10	>10	>10
**27**	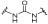	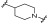	0.34 ± 0.01	>10	>10

**Table 4:** SAR of the R3 position.

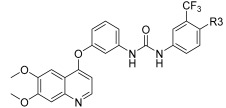				
Compd.	R3	BaF3-TEL-c-KIT (GI_50_: μM)	BaF3-TEL-c-KIT-T670I (GI_50_: μM)	BaF3 (GI_50_: μM)
**22c**	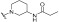	0.28 ± 0.025	0.92 ± 0.04	1.3 ± 0.17
**22d**	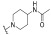	0.11 ± 0.016	0.046 ± 0.001	7.1 ± 0.38
**22e**	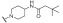	0.43 ± 0.01	0.34 ± 0.025	>10
**22f**	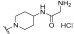	0.13 ± 0.01	0.58 ± 0.02	5 ± 1.0
**22g**	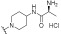	0.12 ± 0.011	0.48 ± 0.011	3 ± 0.15
**22h**	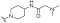	0.038 ± 0.001	0.053 ± 0.001	2.9 ± 0.17
**22i**	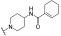	0.76 ± 0.0057	0.76 ± 0.1	1.1 ± 0.47
**22j**	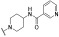	0.02 ± 0.001	0.32 ± 0.025	7 ± 0.74
**22k**	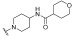	0.087 ± 0.0015	0.083 ± 0.021	2.9 ± 0.056
**22l**	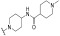	0.16 ± 0.01	0.081 ± 0.006	1.7 ± 01
**22m**	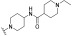	0.039 ± 0.041	0.087 ± 0.008	1.3 ± 0.11
**22n**	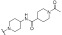	0.059 ± 0.001	0.23 ± 0.0001	3.4 ± 1.2
**22o**	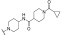	0.13 ± 0.0057	0.12 ± 0.02	0.8 ± 0.084
**22p**	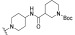	0.13 ± 0.001	0.17 ± 0.05	0.51 ± 0.15
**22q**	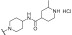	0.46 ± 0.021	0.65 ± 0.076	4.0 ± 0.32
**22r**	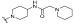	0.12 ± 0.0001	0.037 ± 0.009	0.48 ± 0.006
**22s**	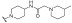	0.33 ± 0.015	0.2 ± 0.011	0.87 ± 0.068
**22t**	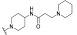	0.44 ± 0.03	0.26 ± 0.052	7.6 ± 0.61
**22u**	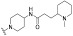	0.19 ± 0.021	0.2 ± 0.02	1.2 ± 0.11
**22v**	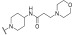	0.042 ± 0.003	0.059 ± 0.006	3.6 ± 0.89
**22w**	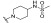	3.9 ± 0.023	3.8 ± 0.02	>10
**22x**	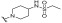	0.034 ± 0.002	0.33 ± 0.029	6.2 ± 0.38
**22y**	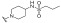	0.059 ± 0.003	0.95 ± 0.04	4.2 ± 0.36
**22z**	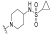	0.14 ± 0.021	0.063 ± 0.002	1.9 ± 0.058
**22aa**	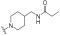	0.057 ± 0.0021	0.063 ± 0.001	>10
**22ab**	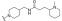	0.25 ± 0.06	0.46 ± 0.01	4.4 ± 1.37

**Table 5:** SAR exploration focused on the L2/R1 moieties.

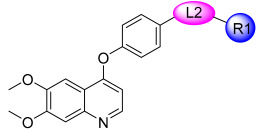					
Compd.	L2	R1	Parental BaF3 (GI_50_: μM)	BaF3-Tel-c-KIT (GI_50_: μM)	BaF3-Tel-c-KIT-T670I (GI_50_: μM)
**CHMFL-KIT-8140**	**-**	**-**	>10	0.057	0.063
**35a**	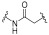	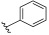	>10	>10	>10
**35b**	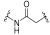	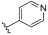	5.13	4.17	6.99
**36**	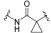	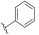	>10	>10	>10
**35c**	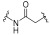	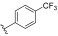	2.16	0.022	0.001
**35d**	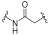	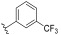	1.89	0.020	0.001
**35e**	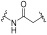	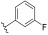	1.0	0.923	0.342
**35f**	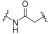	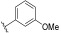	2.78	0.293	0.339
**35g**	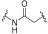	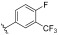	1.03	0.024	0.013
**35h**	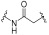	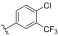	5.97	0.001	0.004
**35i**	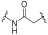	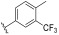	7.26	0.017	0.014
**35j**	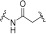	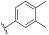	6.42	0.027	0.015
**35k**	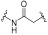	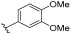	4.13	0.319	0.179
**35l**	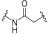	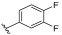	0.67	0.312	0.309
**35m**	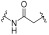	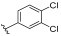	2.03	0.342	0.037
**35n**	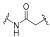	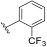	6.70	0.424	0.309
**35o**	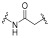	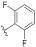	1.93	2.78	1.70
**35p**	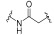	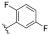	0.704	0.040	0.285
**35q**	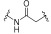	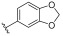	3.10	0.11	0.167
**35r**	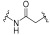	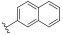	6.23	0.112	0.034
**35s**	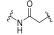	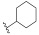	2.47	0.635	0.362
**35t**	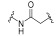	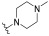	8.70	7.33	8.52
**43**	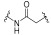	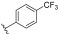	6.18	2.90	0.668
**44**	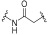	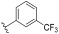	10	2.35	4.61

**Table 6:** SAR exploration focused on the substitution (L1) moiety.

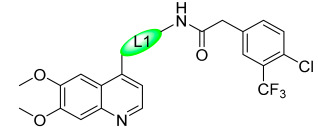				
Compd.	L1	Parental BaF3 (GI_50_: μM)	BaF3-Tel-c-KIT (GI_50_: μM)	BaF3-Tel-c-KIT-T670I (GI_50_: μM)
**48**	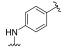	2.21	0.742	0.4
**50**	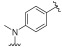	2.67	4.28	8.46
**52**	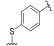	3.09	0.154	0.11
**39**	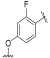	3.21	0.049	0.018
**35u**	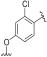	8.22	0.081	0.070
**40a**	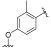	4.86	0.061	0.075
**40**	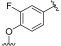	1.99	0.116	0.021
**40b**	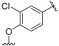	9.45	0.078	0.056
**40c**	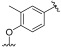	7.05	0.105	0.009
**40d**	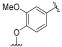	8.48	0.034	1.56
**40e**	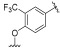	2.92	3.0	2.84
**40f**	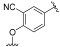	9.97	0.989	0.21

**Table 7:** SAR exploration of the linker moiety (L).

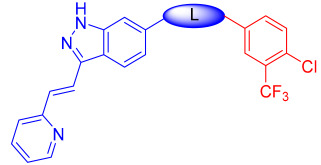				
Compd.	Linker (L)	BaF3 (GI_50_: μM)	BaF3-Tel-c-KIT (GI_50_: μM)	BaF3-Tel-c-KIT-T670I (GI_50_: μM)
**Axitinib**	-	1.64	0.105	0.108
**57a**	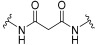	7.4	0.025	0.002
**58a**	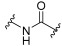	1.71	2.97	1.71
**58b**	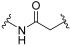	3.79	5.5	3.18
**59**	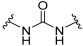	1.9	1.6	1.15
**57b**	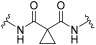	>10	1	0.23

**Table 8:** SAR exploration of the R1moiety.

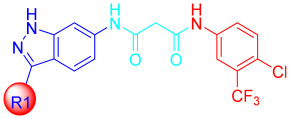				
Compd.	R1	BaF3 (GI_50_: μM)	BaF3-Tel-c-KIT (GI_50_: μM)	BaF3-Tel-c-KIT-T670I (GI_50_: μM)
**67a**	H	>10	>10	2.67
**67b**	Me	>10	>10	1.88
**67c**	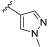	>10	0.35	0.023
**67d**	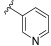	>10	0.32	0.045
**67e**	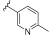	4.91	0.183	0.059
**67f**	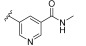	>10	>10	4.48
**67g**	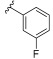	>10	1.12	0.179
**67h**	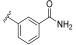	>10	1.55	0.339
**67i**	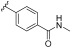	>10	0.338	0.057
**67j**	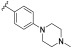	1.35	0.025	0.012
**67k**	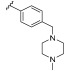	0.911	0.019	0.012

**Table 9:** SAR exploration of the R2 position.

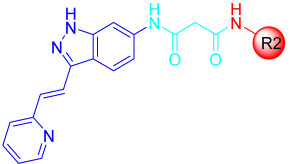				
Compd.	Tail (R2)	BaF3 (GI_50_: μM)	BaF3-Tel-c-KIT (GI_50_: μM)	BaF3-Tel-c-KIT-T670I (GI_50_: μM)
**57c**	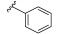	6.72	2.22	0.117
**57d**	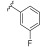	3.97	1.15	0.044
**57e**	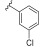	3.61	0.923	0.111
**57f**	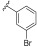	6.09	0.943	0.113
**57g**	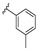	>10	1.43	0.17
**57h**	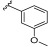	>10	2.74	0.234
**57i**	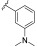	>10	3.87	0.594
**57j**	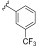	>10	0.216	0.086
**57k**	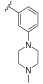	>10	>10	6.17
**57l**	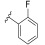	5.09	2.16	0.312
**57m**	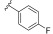	5.86	0.554	0.111
**57n**	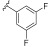	4.24	1.87	0.399
**57o**	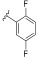	5.08	3.46	0.847
**57p**	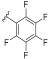	3.68	2.95	1.3

**Table 10:** IC_50_ determination of trisubstituted derivatives.

Compd.	R1	R2	R3	R4	IC_50_ [nM]			
					KIT^WT^	KIT^V559D/T670I^	KIT^V559D/V654A^	KIT^D816H^
**70a**	I	CF_3_	H	CH_3_	6.8 ± 21	a	a	a
**70b**	H	CF_3_	F	CH_3_	555.7 ± 41.6	a	a	a
**70c**	I	CF_3_	Et	CH_3_	1039.3 ± 513.9	a	a	a
**70d**	I	CF_3_	F	CH_3_	183.7 ± 186.5	a	a	a
**71a**	H	CF_3_	H	CH_3_	230.7 ± 1.2	a	a	a
**71b**	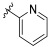	CF_3_	H	CH_3_	3.5 ± 0.6	116 ± 20.1	a	a
**71c**	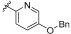	CF_3_	H	CH_3_	120.9 ± 154.7	a	a	a
**71d**	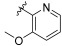	CF_3_	H	CH_3_	6.7 ± 1.9	158.7 ± 43	a	a
**71e**	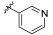	CF_3_	H	CH_3_	2.1 ± 0.6	24 ± 3.1	1101.6 ± 47.8	137.6 ± 2.3
**71f**	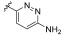	CF_3_	H	CH_3_	5.8 ± 0.2	150.2 ± 35.3	a	2267.8 ± 105.3
**71g**	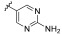	CF_3_	H	CH_3_	1.9 ± 0.6	21.4 ± 1.2	246.6 ± 71.2	42.2 ± 13.0
**71h**	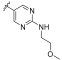	CF_3_	H	CH_3_	3.3 ± 0.9	106.8 ± 7.2	252.5 ± 13.2	2614.3 ± 745.4
**71i**	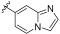	CF_3_	H	CH_3_	1.6 ± 0.9	72.0 ± 5.2	a	a
**71j**	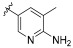	CF_3_	H	CH_3_	2 ± 0.5	29.8 ± 1.4	777.2 ± 88.6	99.3 ± 43.3
**71k**	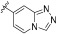	CF_3_	H	CH_3_	2.5 ± 0.9	2147.2 ± 213.1	a	a
**71l**	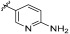	CF_3_	H	CH_3_	2.0 ± 0.6	28.8 ± 1.3	908.3 ± 165.0	140 ± 13.4
**71m**	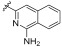	CF_3_	H	CH_3_	4 ± 0	18.2 ± 6.8	313.5 ± 132.2	104.3 ± 8.7
**71n**	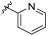	H	H	CH_3_	239.6 ± 307.6	a	a	a
**71o**	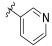	H	H	CH_3_	3.3 ± 1.8	a	a	a
**71p**	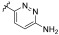	H	H	CH_3_	100.3 ± 84.2	a	a	a
**71q**	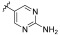	H	H	CH_3_	2.6 ± 1.0	a	a	a
**71r**	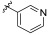	F	H	CH_3_	2.3 ± 0.5	a	a	a
**71s**	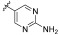	F	H	CH_3_	6.5 ± 1.1	a	a	1706.6 ± 803.4
**71t**	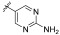	CF_3_	CH_3_	H	6.2 ± 2.3	21 ± 3.6	1084.7 ± 304.0	325.8 ± 111.1
Ponatinib					1.7 ± 0.7	17.4 ± 9.8	136.0 ± 39.9	20 ± 2.2

^a^ No inhibition.

**Table 11:** GI_50_ determination of trisubstituted derivatives.

Compd.	GI_50_ [nM]				
	GIST-48B	GIST-T1	GIST-T1-T670I	GIST-T1-D816E	GIST430-V654A
**70a**	4877 ± 358	896 ± 175	3342 ± 282	9891 ± 1741	3905 ± 1799
**70b**	a	10,019 ± 1870	15,291 ± 2665	a	a
**70c**	9942 ± 1049	11,006 ± 825	7609 ± 286	a	15,858 ± 498
**70d**	3627 ± 1346	5729 ± 705	9512 ± 403	a	a
**71a**	a	7840 ± 891	10,136 ± 2058	a	a
**71b**	3627 ± 532	55 ± 9	97 ± 19	1673 ± 328	697 ± 76
**71c**	1860 ± 250	121 ± 320	4375 ± 1985	a	2005 ± 890
**71d**	3793 ± 525	82 ± 115	165 ± 33	991 ± 95	564 ± 32
**71e**	2705 ± 296	51 ± 13	76 ± 18	332 ± 77	333 ± 1
**71f**	6247 ± 242	54 ± 11	89 ± 17	580 ± 141	459 ± 47
**71g**	2024 ± 545	23 ± 6	44 ± 8	141 ± 12	51 ± 5
**71h**	5132 ± 511	119 ± 21	198 ± 25	704 ± 110	481 ± 6
**71i**	3037 ± 620	59 ± 12	184 ± 26	2289 ± 382	723 ± 45
**71j**	2087 ± 147	59 ± 11	117 ± 29	474 ± 37	307 ± 2
**71k**	4146 ± 1116	282 ± 78	2971 ± 1150	a	4145 ± 94
**71l**	2237 ± 156	42 ± 8	67 ± 12	221 ± 42	404 ± 335
**71m**	2065 ± 288	191 ± 64	301 ± 68	645 ± 744	926 ± 385
**71n**	a	7097 ± 522	12,473 ± 3527	a	a
**71o**	a	150 ± 37	6352 ± 1825	9610 ± 1089	4032 ± 97
**71p**	a	4189 ± 727	a	a	a
**71q**	6877 ± 1409	33 ± 6	1224 ± 758	1269 ± 53	2063 ± 1294
**71r**	11,966 ± 2280	57 ± 15	3294 ± 314	5358 ± 1309	1639 ± 176
**71s**	8285 ± 2983	31 ± 16	1362 ± 576	904 ± 490	920 ± 105
**71t**	15,832 ± 390	35 ± 3	51 ± 1	381 ± 33	308 ± 26
Ponatinib	2000 ± 450	17 ± 8	40 ± 19	106 ± 60	149 ± 36

^a^ No inhibition.

**Table 12:** SAR of the literature PDGFR inhibitors in KIT-mutant Ba/F3 cell lines and their effect on KDR.

Compd.	Structure	Ba/F3 GI_50_ (μM)				
		Parental	Exon 11 Del + V654A	Exon 11 Del + D816H	Exon 11 Del + T670I	KDR
**AZD2932**	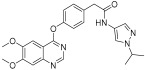	>10	0.012	0.070	0.004	0.093
**I-a**	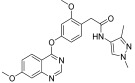	4.289	0.685	0.786	1.918	2.674
**I-b**	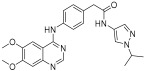	>10	0.337	2.592	0.078	3.595
**I-c**	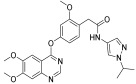	>10	0.008	0.026	0.012	0.442
**I-d**	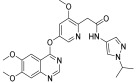	>10	0.021	0.176	0.060	8.030
**I-e**	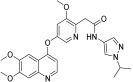	>10	0.013	0.027	0.061	3.924
**I-f**	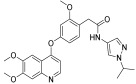	>10	0.006	0.008	0.007	0.077
**II**	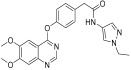	>10	0.044	0.488	0.007	0.680
**II-a**	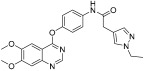	>10	0.021	0.250	0.141	1.615
**II-b**	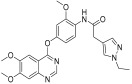	>10	0.078	0.251	0.086	2.551
**III**	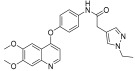	>10	0.026	0.047	0.007	0.082
**III-a**	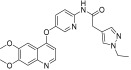	>10	0.150	0.518	0.008	0.761
**IV**	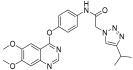	>10	0.003	0.019	0.017	0.612
**V**	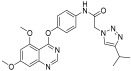	>10	0.007	0.050	0.055	1.222
**V-a**	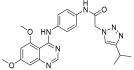	>10	0.003	0.048	0.107	5.408
**V-b**	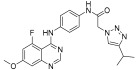	>10	0.002	0.025	0.068	>10
**75**	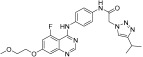	>10	0.003	0.009	0.016	1.378

**Table 13:** c-KIT enzymatic inhibitory activity and GIST-T1 cell proliferation with the synthesized compounds.

Compd.	Structure	IC_50_ (nM)	GI_50_ (nM)
		c-KIT	GIST-T1
**81a**	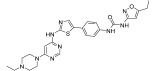	82	2.2
**81b**	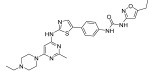	97	3.2
**81c**	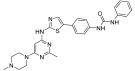	20	5.4
**81d**	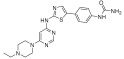	98	86
**81e**	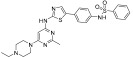	54	42
**81f**	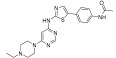	130	25
**81g**	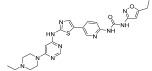	43	1.0
**90**	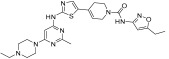	82	4.9
**93**	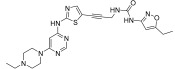	38	9.9
**95**	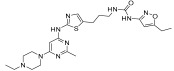	50	10
**81h**	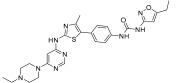	122	3.3
**84**	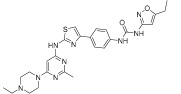	950	>1000
**81i**	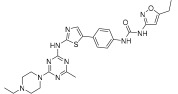	86	13
**81j**	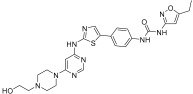	71	3.3
**81k**	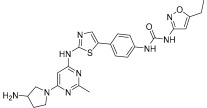	15	6.4
**Sunitinib**	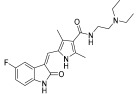	48	38

**Table 14:** Thiazole analogs for enzyme inhibition and cell proliferation.

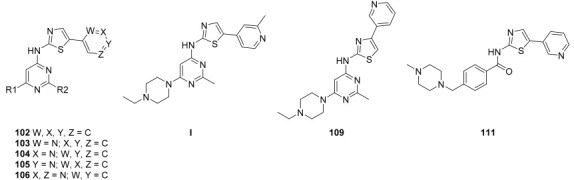						
Compd.	R1	R2	IC_50_ (nM)		GI_50_ (nM)	
			c- KIT	wt-FLT3	GIST-T1	AML MOLM-13
**I**	-	-	97	38	3.2	2.0
**102**	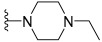	-CH_3_	24	38	8.0	35
**103**	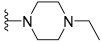	-CH_3_	100	24	42	36
**104a**	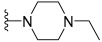	-CH_3_	69	63	26	140
**104b**	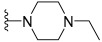	-H	91	60	80	81
**105a**	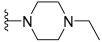	-CH_3_	56	30	7.1	9.4
**105b**	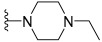	-H	29	20	15	12
**105c**	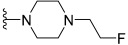	-CH_3_	47	37	3.8	7.7
**105d**	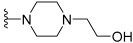	-CH_3_	49	43	2.8	6.5
**105e**	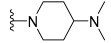	-CH_3_	53	35	1.5	3.5
**105f**	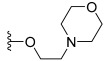	-CH_3_	49	35	27	42
**105g**	-	-	100	64	7.7	39
**106**	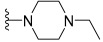	-CH_3_	123	163	120	280
**109**	-	-	>1000	>1000	>1000	>1000
**111**	-	-	107	103	647	544
**imatinib**			53	131	40	>1000
**sunitinib**			48	31	38	54
**regorafenib**			116	82	119	887
**midostaurin**			109	40	235	68

**Table 15:** Structures and kinase inhibitory activities of compounds **116a**–**122b**.

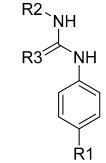						
Compd.	R1	R2	R3	IC_50_ (nM)		
				PDGFRα	VEGFR2	FGFR1
**I**	** 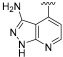 **	** 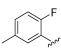 **	O	15	250	5479
**116a**	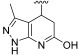	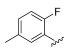	O	>50,000	>50,000	33,849
**116b**	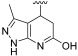	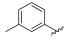	O	>50,000	>50,000	18,325
**119a**	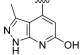	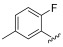	O	1179	1856	4635
**119b**	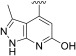	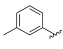	O	560	1224	2264
**122c**	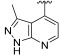	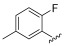	O	87	4293	>50,000
**122d**	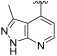	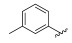	O	40	1225	>50,000
**122e**	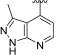	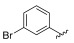	O	137	3145	>50,000
**122f**	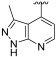	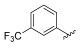	O	106	1921	>50,000
**122a**	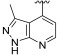	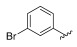	S	1081	6235	19,999
**122b**	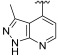	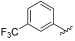	S	1572	8008	42,859

**Table 16:** Kinase spectrum of **122d** against RTK.

Compd.	IC_50_ (nM)							
	PDGFRα	VEGFR2	FGFR1	c-KIT	PDGFRβ	FLT3	VEGFR1	VEGFR3
**122d**	40	1225	>50,000	2.1	197	1880	3493	376

**Table 17:** Inhibitory activities of compounds **126a**–**122i** against kinases.

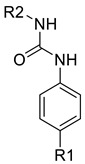					
Compd.	R1	R2	IC_50_ (nM)		
			c-KIT	PDGFRα	VEGFR2
**126a**	** 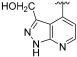 **	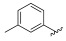	2.4	87	4920
**126b**	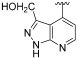	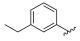	3.7	32	3360
**122g**	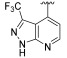	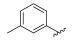	>50,000	>50,000	>50,000
**122h**	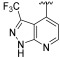	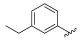	6768	>50,000	>50,000
**122i**	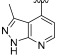	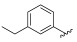	3.9	22	1208

**Table 18:** Inhibitory activities of compounds **122j–n**, **130a–m**, and **127a–y** against kinases.

Compd.	R2	IC_50_ (nM)			Compd.	R2	IC_50_ (nM)		
		c-KIT	PDGFRα	VEGFR2			c-KIT	PDGFRα	VEGFR2
**122j**	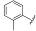	10,890	305	4322	**127e**	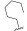	1.4	27	10,462
**122k**	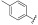	1831	65	5613	**127f**	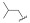	320	1123	8634
**122l**	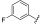	4794	262	6228	**127g**	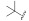	1416	1465	11,382
**122m**	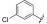	2402	182	4050	**127h**	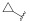	553	1133	4523
**122n**	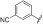	201	21,400	>50,000	**127i**	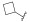	318	732	5002
**130a**	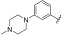	19	215	6235	**127j**	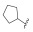	144	576	6229
**130b**	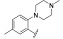	1949	1779	3940	**127k**	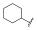	>50,000	>50,000	>50,000
**130c**	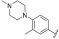	7.2	127	3695	**127l**	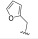	51	387	3503
**130d**	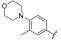	2.4	7.2	2280	**127m**	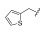	26	157	1791
**130e**	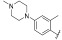	2225	1101	6816	**127n**	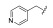	78	472	4079
**130f**	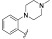	4581	928	4164	**127o**	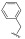	46,064	>50,000	>50,000
**130g**	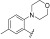	397	18,925	26,163	**127p**	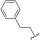	3.7	82	9760
**130h**	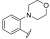	6438	4077	36,890	**127q**	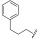	1.7	24	3857
**130i**	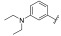	12	136	41,966	**127r**	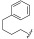	6.2	135	10,917
**130j**	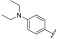	12	176	5352	**127s**	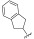	8.7	83	>50,000
**130k**	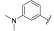	2.6	83	6920	**127t**	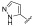	120	411	2391
**130l**	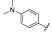	9.3	309	9046	**127u**	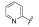	20	401	>50,000
**130m**	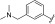	22,056	4608	15,935	**127v**	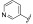	14	313	3109
**127a**	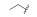	38,667	>50,000	>50,000	**127w**	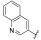	1.9	22	1845
**127b**	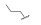	21,432	>50,000	>50,000	**127x**	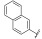	20	124	>50,000
**127c**	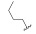	20,556	43,877	>50,000	**127y**	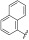	3.6	97	>50,000
**127d**	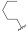	3.5	164	>50,000					

**Table 19:** SAR overview for 3-position imidazopyridine modifications.

Compd.	Structure	IC_50_ [nm]	Cell IC_50_ [nm]	Cl_int_ HLM/MLM	Solubility [µM]
**I**	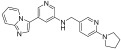	12	20	h/h	101
**II**	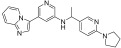	83	2600	h/h	124
**III**	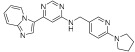	5.4	429	m/h	<3
**IV**	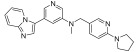	240	-	-	8
**V**	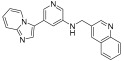	>10,000	-	m/m	-
**VI**	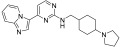	>10,000	-	m/m	-
**VII**	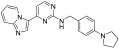	5.6	680	-	7
**VIII**	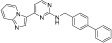	15	340	m/m	<3

**Table 20:** SAR overview for six-position imidazopyridine modifications.

Compd.	Structure	IC_50_ [nm]	Cell IC_50_ [nm]	Cl_int_ HLM/MLM	Solubility [µM]
**IX**	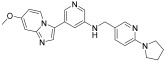	13	300	h/h	81
**X**	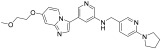	12	110	h/h	9
**XI**	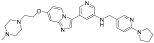	11	290	m/h	100
**XII**	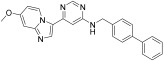	4.6	160	m/L	<2
**XIII**	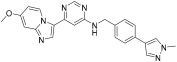	8.6	55	m/L	<2
**135a**	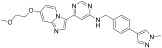	7.3	48	m/h	<2

**Table 21:** SAR overview of advanced c-KIT inhibitors.

Compd.	Cell IC_50_ (nM)	hHEP [µL/min/10^6^ cells]	CACO-2 [10^6^ cm/s] (Efflux)	FaSSiF [µg/mL]	hERG [%inh] at 10 µM	LogD	pK_a_
**135a**	48	18	12 (0.9)	2	1%	3.8	-
**136a**	78	8	9.5 (0.7)	4	-	4.0	-
**136b**	46	4	6.4 (0.8)	-	-	3.0	-
**135b**	93	16	3.1 (4.7)	16	−15%	4.4	6.9
**135c**	61	<4	5.7 (57)	97	−59%	1.9	9.5
**135d**	43	-	-	-	−86%	2.5	nd
**135e**	70	18	12 (5.4)	-	−78%	2.9	9.1
**135f**	75	9	21 (5.0)	13	−23%	3.0	-
**135g**	76	<4	7.6 (18)	10	−8%	2.8	5.8
**135h**	56	10	9.5 (15)	16	−19%	1.5	7.3
**135i**	73	10	1.8 (1.3)	2	−4%	4.3	8.3
**137**	54	6	7.6 (7.5)	10	−7%	3.2	6.3
**135j**	59	21	5.7 (14)	374	−86%	2.6	9.7

**Table 22:** c-KIT enzymatic inhibitory activities of thiazolo[5,4-*b*]pyridine derivatives.

Compd.	R1	R2	IC_50_ (µM) ^a^	Entry	R1	R2	IC_50_ (µM) ^a^
**imatinib**	-	-	0.27	**142p**	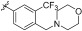	-	5.72
**sunitinib**	-	-	0.14	**142q**	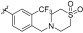	-	3.23
**142a**	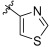	-	inactive ^b^	**142r**	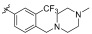	-	0.14
**142b**	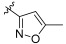	-	inactive ^b^	**142s**	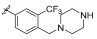	-	0.37
**142c**	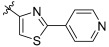	-	inactive ^b^	**142t**	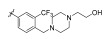	-	1.25
**142d**	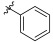	-	inactive ^b^	**142u**	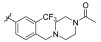	-	4.56
**142e**	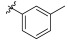	-	inactive ^b^	**142v**	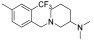	-	0.39
**142f**	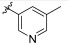	-	inactive ^b^	**142w**	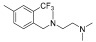	-	0.25
**142g**	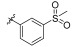	-	inactive ^b^	**143a**	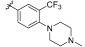	cyclohexyl	1.51
**142h**	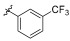	-	9.87	**143b**	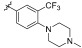	phenyl	0.74
**142i**	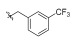	-	inactive ^b^	**143c**	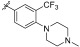	methyl	0.1
**142j**	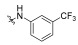	-	inactive ^b^	**143d**	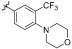	cyclohexyl	inactive ^b^
**142k**	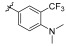	-	4.31	**143e**	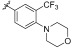	methyl	0.88
**142l**	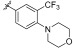	-	1.76	**143f**	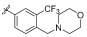	methyl	3.63
**142m**	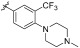	-	2.17	**143g**	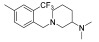	methyl	0.18
**142n**	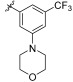	-	inactive ^b^	**143h**	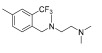	methyl	0.10
**142o**	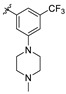	-	5.03				

^a^ Radiometric biochemical kinase assay results. ^b^ less than 50% inhibition at a concentration of 10 µM.

**Table 23:** Antiproliferative activities of thiazolo[5,4-*b*]pyridine derivatives.

Compd.	GI_50_ (µM) ^a^		Entry	GI_50_ (µM) ^a^	
	GIST-T1	HMC1.2		GIST-T1	HMC1.2
**imatinib**	0.02 ± 0.01	27.10 ± 3.36	**142p**	0.66 ± 0.10	8.55 ± 2.40
**sunitinib**	0.01 ± 0.00	2.53 ± 0.35	**142q**	0.42 ± 0.07	15.53 ± 3.97
**142a**	>50	Inactive ^b^	**142r**	0.01 ± 0.00	1.15 ± 0.96
**142b**	33.05 ± 7.09	Inactive ^b^	**142s**	0.02 ± 0.00	1.33 ± 0.43
**142c**	33.69 ± 3.45	Inactive ^b^	**142t**	0.12 ± 0.03	6.62 ± 0.75
**142d**	16.36 ± 2.73	25.18 ± 2.21	**142u**	0.66 ± 0.08	11.31 ± 0.34
**142e**	3.45 ± 0.41	11.53 ± 0.62	**142v**	0.05 ± 0.01	6.81 ± 0.67
**142f**	20.94 ± 2.00	Inactive ^b^	**142w**	0.02 ± 0.01	4.99 ± 0.43
**142g**	10.84 ± 1.06	36.77 ± 10.9	**143a**	0.08 ± 0.01	4.58 ± 0.32
**142h**	0.96 ± 0.10	7.26 ± 4.13	**143b**	0.10 ± 0.01	5.22 ± 0.59
**142i**	7.16 ± 0.87	9.65 ± 1.06	**143c**	0.01 ± 0.00	1.52 ± 0.43
**142j**	3.31 ± 0.73	6.35 ± 0.32	**143d**	8.13 ±1.81	Inactive ^b^
**142k**	0.18 ± 0.03	2.27 ± 0.84	**143e**	0.23 ± 0.07	14.04 ± 10.2
**142l**	0.08 ± 0.01	4.34 ± 1.31	**143f**	0.30 ± 0.06	28.39 ± 13.3
**142m**	0.27 ± 0.17	3.41 ± 1.49	**143g**	0.07 ± 0.01	4.88 ± 1.18
**142n**	0.55 ± 0.09	7.00 ± 2.68	**143h**	0.02 ± 0.00	4.98 ± 0.32
**142o**	0.28 ± 0.08	2.36 ± 0.58			

^a^ Radiometric biochemical kinase assay results. ^b^ less than 50% inhibition at a concentration of 10 µM.

## Data Availability

Data is contained within the article and Supplementary Material.

## Supplementary Materials

The following supporting information can be downloaded at: https://www.mdpi.com/article/10.3390/ijms24119450/s1. References [34,35,36,37,38,39] are cited in the Supplementary Materials.
