# Supplementary material for: Small Molecule c-KIT Inhibitors for the Treatment of Gastrointestinal Stromal Tumors: A Review on Synthesis, Design Strategies, and Structure–Activity Relationship (SAR)
Source: Int J Mol Sci. 2023 May 29;24(11):9450. doi: 10.3390/ijms24119450 (PMC10253927; doi:10.3390/ijms24119450)
Supplement: Supplementary file 1 [file ijms-24-09450-s001.zip › ijms-2348368-supplementary.pdf]

Small Molecule c-KIT Inhibitors for the Treatment of Gastrointestinal Stromal Tumors: A Review on Synthesis, Design Strategies, and Structure–Activity Relationship (SAR)

Sreenivasulu Godesi [1], Joohan Lee [1], Hossam Nada [1], Guofeng Quan [1], Ahmed Elkamhawy [1,2], Yongseok Choi [3] and Kyeong Lee [1,*]

[1] BK21 FOUR Team and Integrated Research Institute for Drug Development, College of Pharmacy, Dongguk University-Seoul, Goyang 10326, Republic of Korea
[2] Department of Pharmaceutical Organic Chemistry, Faculty of Pharmacy, Mansoura University, Mansoura 35516, Egypt
[3] College of Biosciences and Biotechnology, Korea University, Seoul 02841, Republic of Korea
* Correspondence: kaylee@dongguk.edu

Supporting Information

Table of Contents

Synthesis and schemes of biologically tested compounds S3−S20

Synthesis of c-KIT Inhibitors Targeting GIST:

Wang et al. designed and synthesized a novel series of substituted *N*-(4-methyl-3-(piperidin-4-yloxy)phenyl) amide derivatives and screened for type-II c-KIT inhibition activity for GISTs against the Tel-c-KIT-BaF3, Parental BaF3, K562 cell lines [1]. The synthesis of the designed compounds was completed in five steps. Etherification of 2-substituted nitrophenols with various Boc-protected R2 groups, hydrogenation of the nitro group, and installation of R3 group *via* amide formation yielded **4**. Removal of the Boc group with HCl and HATU-mediated coupling of the resultant amine with various acids furnished compounds **6a–v** (Scheme S1). Deprotection of the Boc group in **2a**, installation of the R1 group *via* amide formation, and then of the nitro group gave **9**. Coupling of the resultant amine with corresponding acids yielded **10a–d** (Scheme S2). Compound **13** was synthesized in three steps from **3a**. The R3 group was introduced via urea formation, followed by installation of the R1 group *via* Boc deprotection and amide formation (Scheme S3).

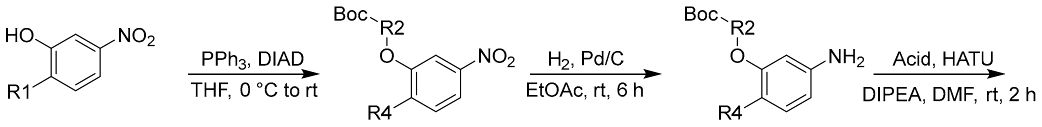

1a-d **2a-f** **3a-f**

1a: R4=Me
1b: R4=Cl
1c: R4=OMe
1d: R4=H

2a-3a: R2=4-piperidine, R4=Me
2b-3b: R2=4-piperidine, R4=Cl
2c-3c: R2=4-piperidine, R4=OMe
2d-3d: R2=4-piperidine, R4=H
2e-3e: R2=3-piperidine, R4=H
2f-3f: R2=ethanamine, R4=H

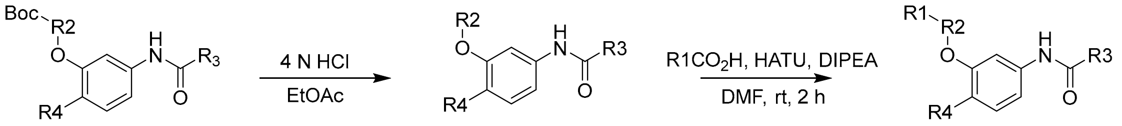

4a-j **5a-j** **6a-v**

4a-5a: R2=4-piperidine, R3=3-CF_3-benzene, R4=Me
4b-5b: R2=4-piperidine, R3=3-CF_3-4-Me-benzene, R4=Me
4c-5c: R2=4-piperidine, R3=3-CF_3-5-F-benzene, R4=Me
4d-5d: R2=4-piperidine, R3=3-CF_3-5-Cl-benzene, R4=Me
4e-5e: R2=4-piperidine, R3=2-(6-CF_3-pyridine), R4=Me
4f-5f: R2=4-piperidine, R3=3-CF_3-benzene, R4=Cl
4g-5g: R2=4-piperidine, R3=3-CF_3-benzene, R4=OMe
4h-5h: R2=4-piperidine, R3=3-CF_3-benzene, R4=H
4i-5i: R2=3-piperidine, R3=3-CF_3-benzene, R4=H
4j-5j: R2=ethanamine, R3=3-CF_3-benzene, R4=Me

6a: R1=nicotinoyl, R2=ethanamine, R3=3-CF_3-benzene, R4=Me
6b: R1=isoxazole-5-formyl, R2=ethanamine, R3=3-CF_3-benzene, R4=Me
6c: R1=nicotinoyl, R2=4-piperidine, R3=3-CF_3-benzene, R4=Me
6d: R1=pyridine-2-formyl, R2=4-piperidine, R3=3-CF_3-benzene, R4=Me
6e: R1=isonicotinoyl, R2=4-piperidine, R3=3-CF_3-benzene, R4=Me
6f: R1=isoxazole-5-formyl, R2=4-piperidine, R3=3-CF_3-benzene, R4=Me
6g: R1=propionyl, R2=4-piperidine, R3=3-CF_3-benzene, R4=Me
6h: R1=acryloyl, R2=4-piperidine, R3=3-CF_3-benzene, R4=Me
6i: R1=nicotinoyl, R2=3-piperidine, R3=3-CF_3-benzene, R4=H
6j: R1=isoxazole-5-formyl, R2=3-piperidine, R3=3-CF_3-benzene, R4=H
6k: R1=nicotinoyl, R2=4-piperidine, R3=3-CF_3-benzene, R4=H
6l: R1=nicotinoyl, R2=4-piperidine, R3=3-CF_3-benzene, R4=Cl
6m: R1=nicotinoyl, R2=4-piperidine, R3=3-CF_3-benzene, R4=OMe
6n: R1=isonicotinoyl, R2=4-piperidine, R3=3-CF_3-4-Me-benzene, R4=Me
6o: R1=nicotinoyl, R2=4-piperidine, R3=3-CF_3-4-Me-benzene, R4=Me
6p: R1=thiazole-5-formyl, R2=4-piperidine, R3=3-CF_3-4-Me-benzene, R4=Me
6q: R1=isoxazole-5-formyl, R2=4-piperidine, R3=3-CF_3-4-Me-benzene, R4=Me
6r: R1=nicotinoyl, R2=4-piperidine, R3=3-CF_3-5-Cl-benzene, R4=Me
6s: R1=isoxazole-5-formyl, R2=4-piperidine, R3=3-CF_3-5-Cl-benzene, R4=Me
6t: R1=nicotinoyl, R2=4-piperidine, R3=3-CF_3-5-F-benzene, R4=Me
6u: R1=nicotinoyl, R2=4-piperidine, R3=2-(6-CF_3-pyridine), R4=Me
6v: R1=isoxazole-5-formyl, R2=4-piperidine, R3=2-(6-CF_3-pyridine), R4=Me

Scheme S1. Synthesis of compounds **6a**–**v**.

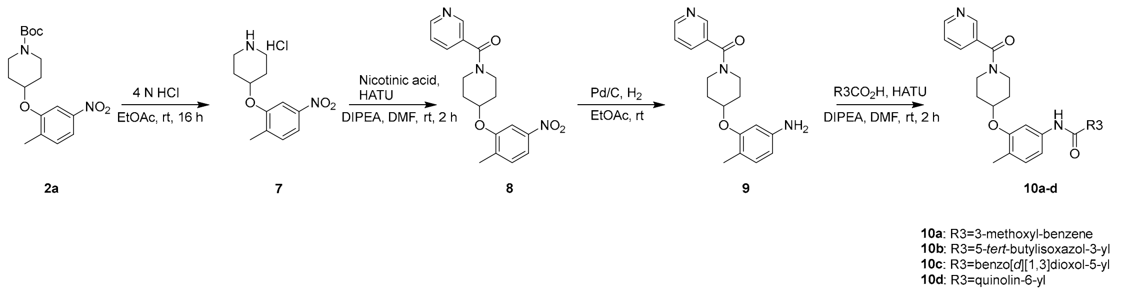

Scheme S2. Synthesis of compounds **10a**–**d**.

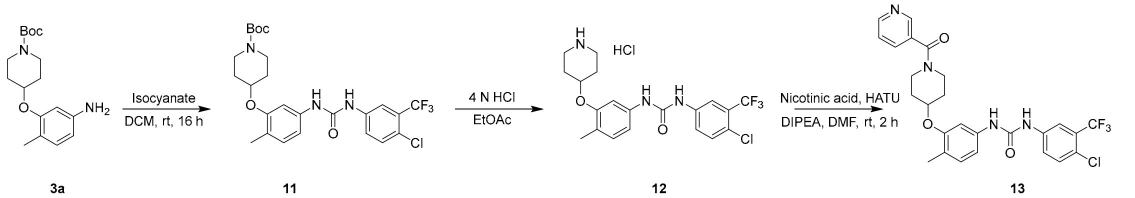

Scheme S3. Synthesis of compound **13**.

Li et al. designed and synthesized a series of 6,7-dimethoxy-4-phenoxyquinoline derivatives and screened for their inhibitory activity against GISTs using wild-type c-KIT and c-KIT T6701 cell lines [2]. Compounds **22a**–**z** and **22aa**–**ab** were synthesized using a multi-step process that involved nucleophilic substitution of 4-chloro-6,7-dimethoxyquinoline with 3-aminophenol to produce compound **16** (Scheme S4). Compound **19** was obtained from Boc-protected 3- or 4-aminopiperidine analogs (**18**) by treating them with chloronitrobenzene derivatives (**17**), followed by urea formation using triphosgene to produce compound **20**. The desired compounds were obtained by Boc-deprotection (**21**) and subsequent treatment of the resultant amine with corresponding carboxylic acids or acyl chlorides. Compound **27** was synthesized through a similar approach with a different reaction order (Scheme S5). The synthesis of compound **33** involved the nucleophilic substitution of Boc-protected 4-aminopiperidine (**18b**) with a trifluoromethyl substituted chloronitrobenzene (**17b**), followed by deprotection of the Boc group (**24**) and connection with **16** through triphosgene. The resulting compound **25** was hydrogenated to provide the amine moiety, which was then treated with propionyl chloride. The next steps involved amide coupling of **16** with 1-(methoxycarbonyl)cyclopropanecarboxylic acid, hydrolysis of the methyl ester **29**, and a coupling reaction of the resultant compound **30** with **19d**. Boc deprotection and acylation were performed to produce compound **33** (Scheme S6).

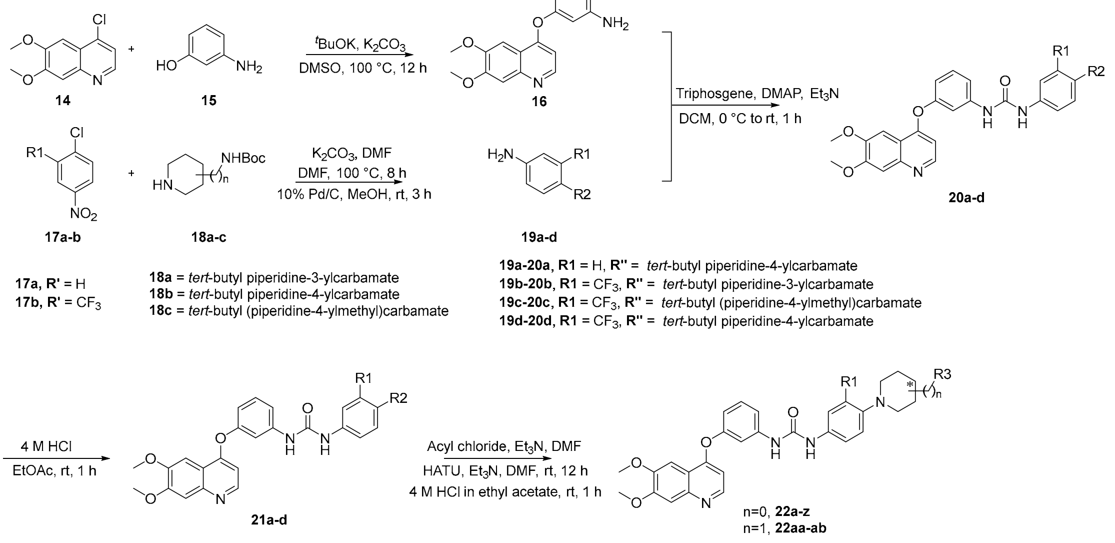

21a, **R1** = H, **R2** = piperidine-4-amine hydrochloride
21b, **R1** = CF_3, **R2** = piperidine-3-amine hydrochloride
21c, **R1** = CF_3, **R2** = piperidine-4-ylmethylamine hydrochloride
21d, **R1** = CF_3, **R2** = piperidine-4-amine hydrochloride

22a, **R1** = CF_3, **R3** = 4-propionamide
22b, **R1** = H, **R3** = 4-propionamide
22c, **R1** = CF_3, **R3** = 3-propionamide
22d, **R1** = CF_3, **R3** = 4-acetamide
22e, **R1** = CF_3, **R3** = 4-(3,3-dimethyl)butanamide
22f, **R1** = CF_3, **R3** = 4-(2-amino)acetamide hydrochloride
22g, **R1** = CF_3, **R3** = 4-[(*S*)-2-amino]propanamide hydrochloride
22h, **R1** = CF_3, **R3** = 4-[2-(dimethylamino)]acetamide
22i, **R1** = CF_3, **R3** = 4-cyclohex-1-enecarboxamide
22j, **R1** = CF_3, **R3** = 4-nicotinamide
22k, **R1** = CF_3, **R3** = 4-tetrahydro-2*H*-pyran-4-caboxamide
22l, **R1** = CF_3, **R3** = 4-(1-methyl)piperidine-4-carboxamide
22m, **R1** = CF_3, **R3** = 4-(1-ethyl)piperidine-4-caboxamide
22n, **R1** = CF_3, **R3** = 4-(1-acetyl)piperidine-4-caboxamide
22o, **R1** = CF_3, **R3** = 4-(1-cyclopropanecarbonyl)piperidine-4-caboxamide
22p, **R1** = CF_3, **R3** = 4-(1-tert butyl carboxylate)piperidine-4-caboxamide
22q, **R1** = CF_3, **R3** = 4-(2-methyl)piperidine-4-carboxamide hydrochloride
22r, **R1** = CF_3, **R3** = 4-[2-(piperidin-1-yl)]acetamide
22s, **R1** = CF_3, **R3** = 4-[2-(3-methylpiperidin-1-yl)]acetamide
22t, **R1** = CF_3, **R3** = 4-[3-(piperidine-1-yl)]propionamide
22u, **R1** = CF_3, **R3** =4-[3-(1-methylpiperidin-2-yl)]propionamide
22v, **R1** = CF_3, **R3** = 4-(3-morpholino)propionamide
22w, **R1** = CF_3, **R3** = 4-methanesulfonamide
22x, **R1** = CF_3, **R3** = 4-ethanesulfonamide
22y, **R1** = CF_3, **R3** = 4-(1-propane)sulfonamide
22z, **R1** = CF_3, **R3** = 4-cyclopropanesulfonamide
22aa, **R1** = CF_3, **R3** = 4-(*N*-propionamide)methanamide
22ab, **R1** = CF_3, **R3** = 4-[*N*-3-(1-methylpiperidin-2-yl)propionamide]methanamide

Scheme S4. Synthesis of compounds **22a**–**z** and **22aa**–**ab**.

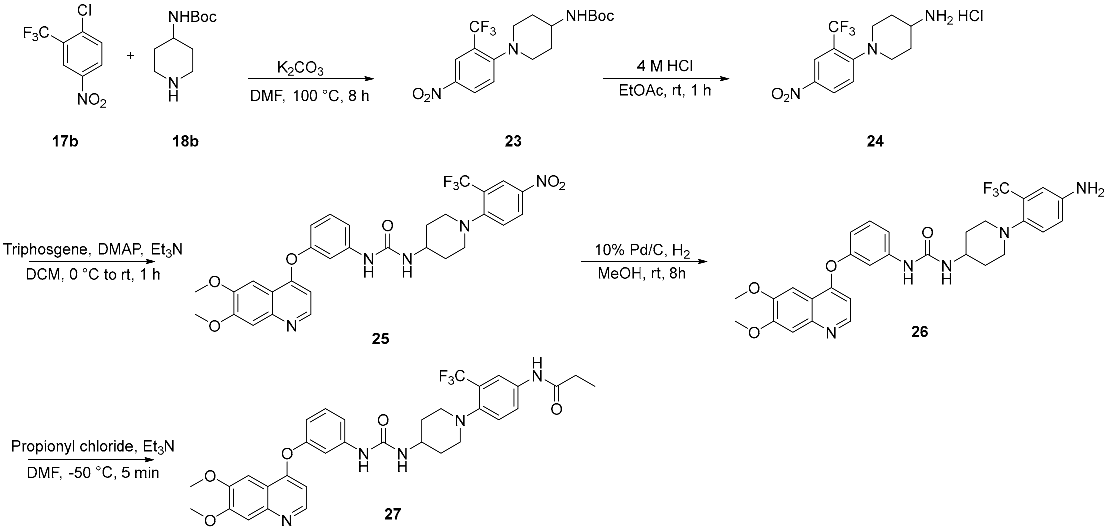

Scheme S5. Synthesis of compound **27**.

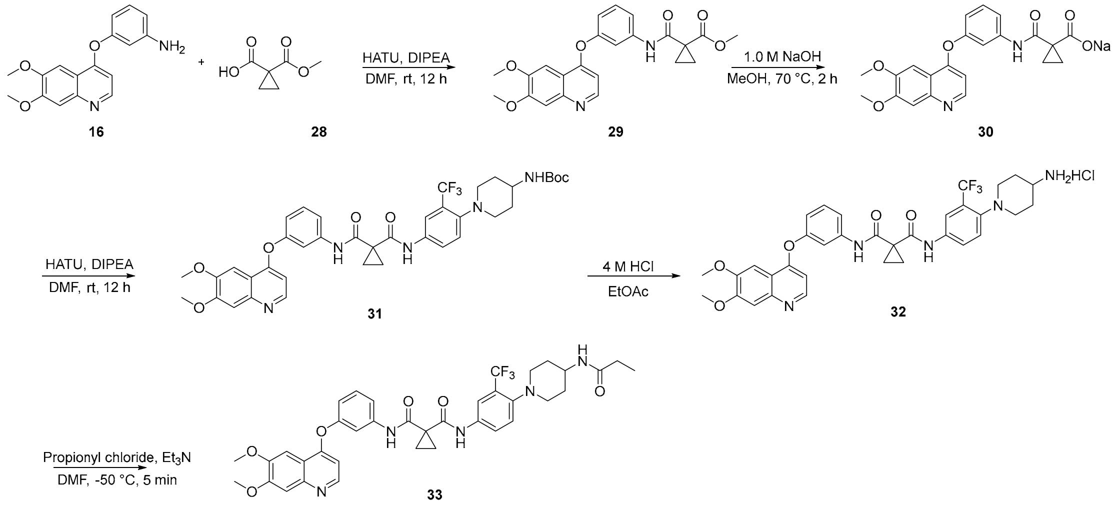

Scheme S6. Synthesis of compound **33**.

In a continued evaluation of their previously developed c-KIT inhibitor **CHMFL-KIT-8140**, Wu et al. designed and synthesized a series of substituted *N*-(4-((6,7-dimethoxyquinolin-4-yl)oxy)phenyl)acetamide derivatives and screened for their inhibitory activity for GISTs against c-KIT kinase and c-KIT T670I mutants [3]. Scheme S7 outlines the synthesis of compounds **35a**–**u**, **36**, and **39**–**40** through a three-step procedure that involves reacting 4-chloro-6,7-dimethoxyquinoline with aminophenols or nitrophenols to obtain quinoline intermediates, followed by reducing the nitro group to an amine using Fe powder in EtOH and coupling the resultant intermediates with substituted phenylacetic acids or acyl chloride to produce the target compounds. Scheme S8 demonstrates the synthetic route to prepare the final products **43** and **44**. The process involves synthesizing intermediate **41** through nucleophilic substitution of methyl 4-hydroxybenzoate with 4-chloro-6,7-dimethoxyquinoline, followed by basic hydrolysis of the ester to yield carboxylic acid **42**. The final products were obtained by amidation of carboxylic acid **42** with substituted phenethyl amine. Scheme S9 illustrates the preparation of quinoline analogues **48** and **50**. The process

involves reacting 4-chloro-6,7-dimethoxyquinoline with 4-nitroaniline to obtain phenylamine derivative **45**, which was then subjected to further methylation to yield intermediate **46**. The nitro groups in **45** and **46** were reduced to give amines **47** and **49**, respectively, which were subsequently coupled with 2-(4-chloro-3-(trifluoromethyl)phenyl)acetic acid to produce the desired quinoline analogs. Scheme S10 outlines the synthesis of compound **52**. Initially, 4-chloro-6,7-dimethoxyquinoline was nucleophilically substituted with 4-aminobenzenethiol to produce amine **51**. This was followed by an amidation reaction of 2-(4-chloro-3-(trifluoromethyl)phenyl)acetic acid with amine **51** to produce the final compound **52**. Scheme S11 shows the synthesis of intermediates **38a–b** *via* nucleophilic substitution of 6,7-dimethoxyquinolin-4-ol with substituted 4-fluoronitrobenzenes, followed by reduction to form aniline intermediates **38c–h**. The desired quinoline compounds **40a–f** were achieved through an amidation reaction of **38c–h** with 2-(4-chloro-3-(trifluoromethyl)phenyl)acetic acid.

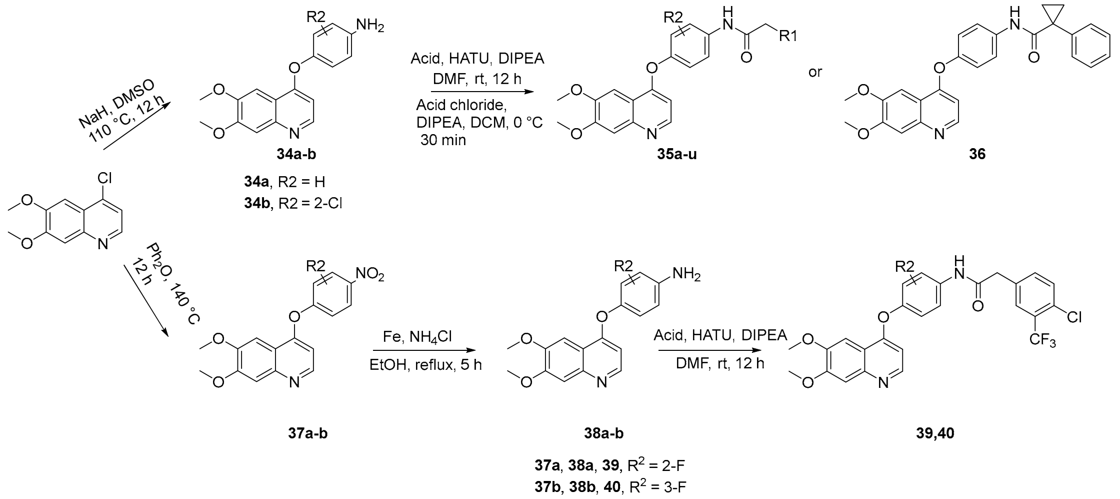

35a, R2 = H, R1 = phenyl
35b, R2 = H, R1 = pyridin-4-yl
35c, R2 = H, R1 = 4-(trifluoromethyl)phenyl
35d, R2 = H, R1 = 3-(trifluoromethyl)phenyl
35e, R2 = H, R1 = 3-fluorophenyl
35f, R2 = H, R1 = 3-methoxyphenyl
35g, R2 = H, R1 = 4-fluoro-3-(trifluoromethyl)phenyl
35h, R2 = H, R1 = 4-chloro-3-(trifluoromethyl)phenyl
35i, R2 = H, R1 = 4-methyl-3-(trifluoromethyl)phenyl
35j, R2 = H, R1 = 3,4-dimethylphenyl
35k, R2 = H, R1 = 3,4-dimethoxyphenyl
35l, R2 = H, R1 = 3,4-difluorophenyl
35m, R2 = H, R1 = 3,4-dichlorophenyl
35n, R2 = H, R1 = 2-(trifluoromethyl)phenyl
35o, R2 = H, R1 = 2,6-difluorophenyl
35p, R2 = H, R1 = 2,4-difluorophenyl
35q, R2 = H, R1 = benzo[*d*][1,3]dioxol-5-yl
35r, R2 = H, R1 = naphthalen-2-yl
35s, R2 = H, R1 = cyclohenxyl
35t, R2 = H, R1 = 4-methylpiperazin-1-yl
35u, R2 = 2-Cl, R1 = 4-chloro-3-(trifluoromethyl)phenyl

Scheme S7. Synthesis of compounds **35a–u**, **36**, and **39–40**.

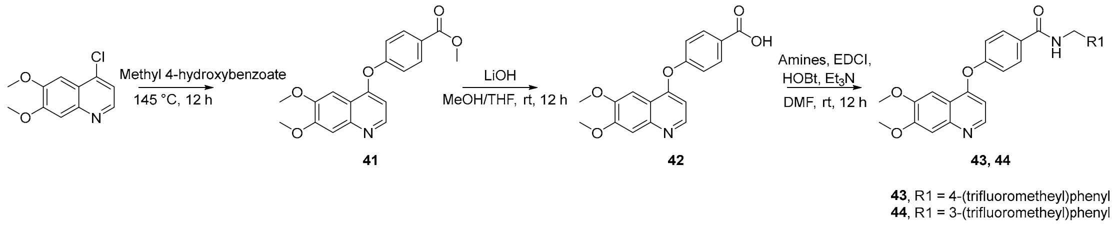

Scheme S8. Synthesis of Compounds **43–44**.

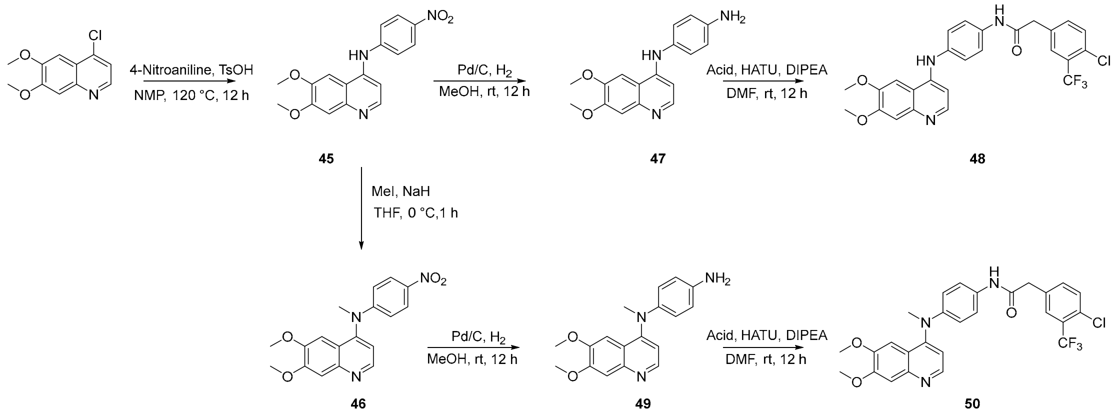

Scheme S9. Synthesis of compounds **48** and **50**.

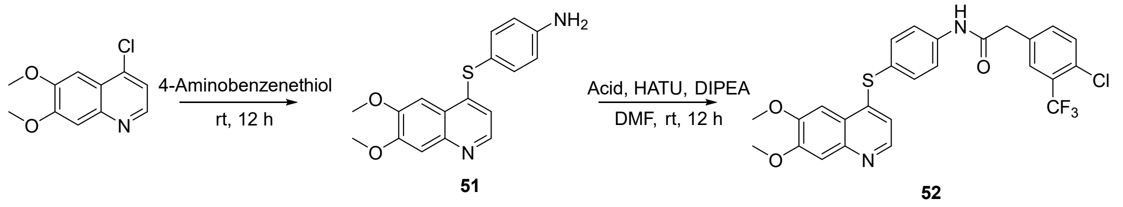

Scheme S10. Synthesis of compound **52**.

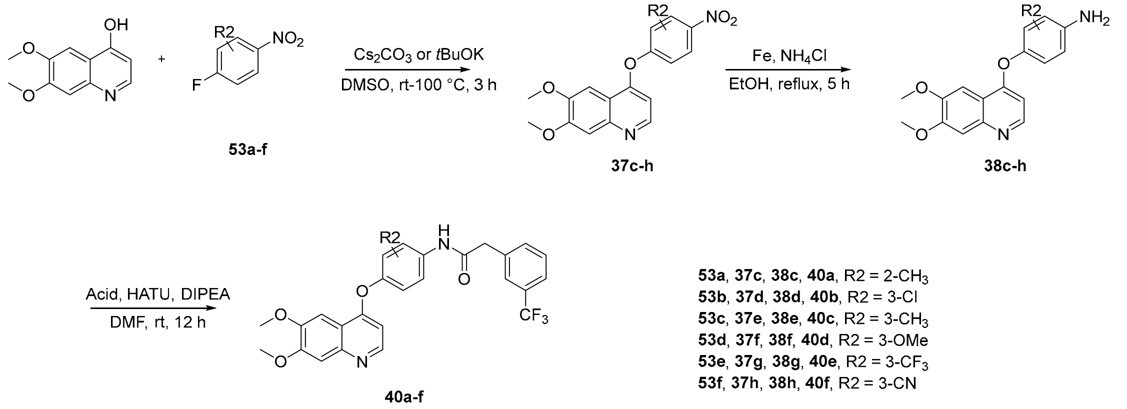

Scheme S11. Synthesis of compounds **40a**–**f**.

Liu et al. designed and synthesized structurally modified derivatives of the FDA-approved drug axitinib and tested them for their inhibitory activity against c-KIT wt and c-KIT-T670I. The synthesis of the designed compounds is illustrated in Schemes S12–S14 [4]. An amide coupling reaction of **54** with carboxylic acid and the basic hydrolysis of the resultant ester **55** yielded carboxylic acid **56**. The synthesis of compounds **57a**–**p** was completed by the amide coupling reaction of the resultant acid with various amines, followed by the deprotection of the THP protective group. HATU-mediated coupling of amine **54** and then conversion of amine **54** to urea, and then removal of the THP group from the resultant intermediates produced **58a**–**b**. A Suzuki cross-coupling reaction of **60** with various boronic acids and THP protection of **61** yielded **62a–k**. Reduction of the nitro group produced amine **63a–k**. Treatment of **64** with methyl hydrogen malonate, followed by ester hydrolysis of **65**, furnished carboxylic acid **66**. Furthermore, an amide coupling reaction of **63a–k** with amine **66**, followed by the cleavage of the THP protective group produced the designed compounds **67a**–**k**.

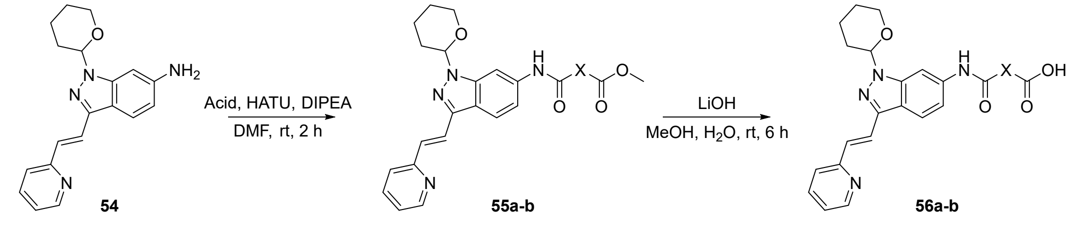

55a-56a, X = CH_2
55b-56b, X = 1,1-cyclopropane

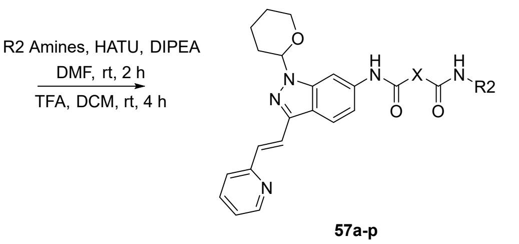

57a, R2 = 4-chloro-3-(trifluoromethyl)phenyl, X = CH_2
57b, R2 = 4-chloro-3-(trifluoromethyl)phenyl, X = 1,1-cyclopropane
57c, R2 = phenyl, X = CH_2
57d, R2 = 3-flurophenyl, X = CH_2
57e, R2 = 3-chlorophenyl, X = CH_2
57f, R2 = 3-bromophenyl, X = CH_2
57g, R2 = m-tolyl, X = CH_2
57h, R2 = 3-(trifluoromethyl)phenyl, X = CH_2
57i, R2 = 3-methoxyphenyl, X = CH_2
57j, R2 = 3-(dimethylamino)phenyl, X = CH_2
57k, R2 = 3-(4-methylpiperazin-1-yl)phenyl, X = CH_2
57l, R2 = 2-fluorophenyl, X = CH_2
57m, R2 = 4-fluorophenyl, X = CH_2
57n, R2 = 3,5-difluorophenyl, X = CH_2
57o, R2 = 2,5-difluorophenyl, X = CH_2
57p, R2 = perfluorophenyl, X = CH_2

Scheme S12. Synthetic route for compounds **57a–p**.

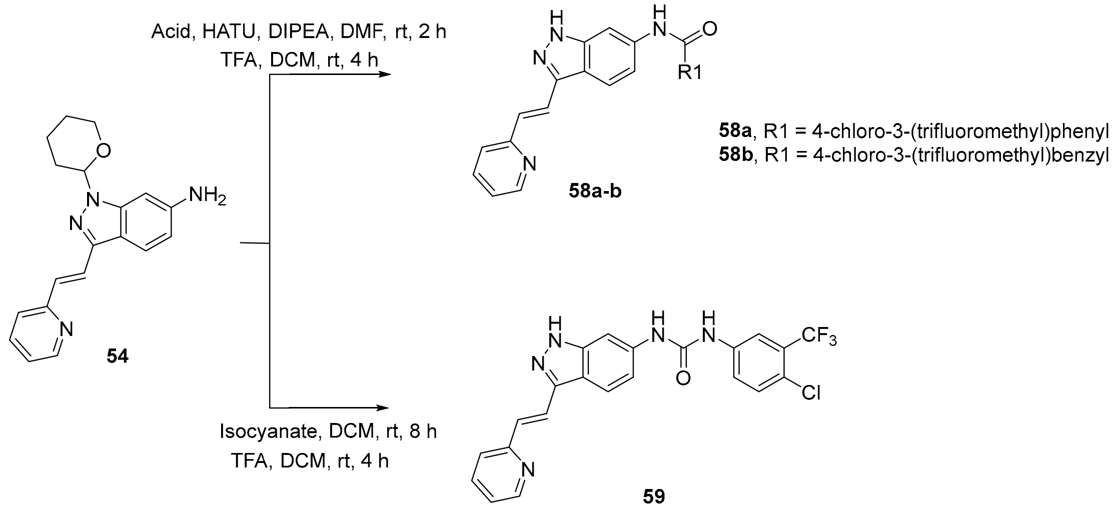

Scheme S13. Synthetic route for compounds **58a–p** and **59**.

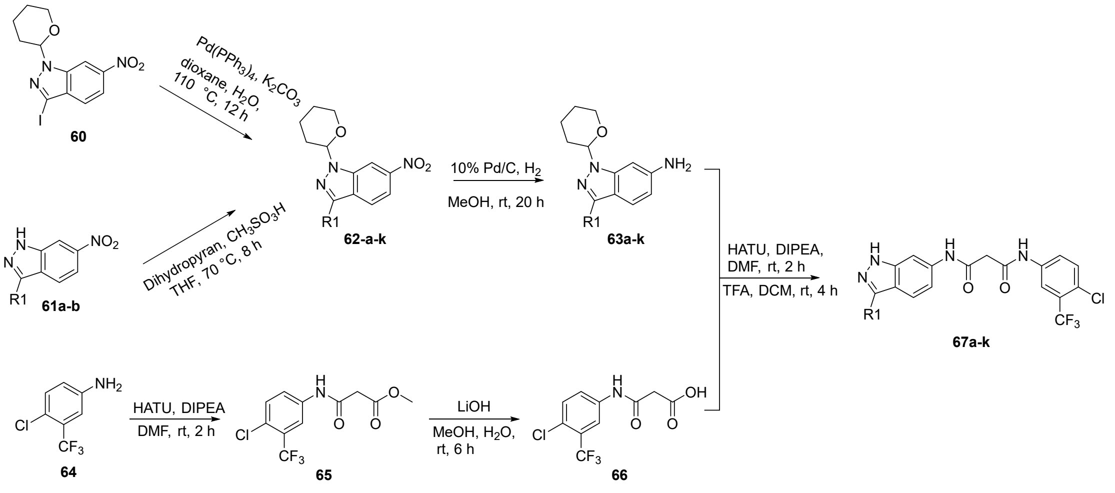

61a-63a, **67a**, R1 = H
61b-63b, **67b**, R1 = Me
61c-63c, **67c**, R1 = methyl-1*H*-pyrazol-4-yl
61d-63d, **67d**, R1 = pyridin-3-yl
61e-63e, **67e**, R1 = 6-methylpyridin-3-yl
61f-63f, **67f**, R1 = 5-(methylcarbamoyl)pyridin-3-yl
61g-63g, **67g**, R1 = 3-fluorophenyl
61h-63h, **67h**, R1 = 3-(3-crarbamoylphenyl)
61i-63i, **67i**, R1 = 3-(4-methylcarbamoyl)phenyl
61j-63j, **67j**, R1 = 3-(4-methylpiperazin-1-yl)phenyl
61k-63k, **67k**, R1 = 3-((4-methylpiperazin-1-yl)methyl)phenyl

Scheme S14. Synthetic route for compounds **67a**–**k**.

Kaitsiotou et al. designed and synthesized trisubstituted 3-ethynyl-*N*-(4-((4-methylpiperazin-1-yl)methyl)phenyl)benzamide derivatives and screened them against various c-KIT mutants such as V654A, T670I, and D816H along with wild-type KIT [5]. The design of compounds was started by maintaining the potency of ponatinib and modifying the substitutions in the R1–R4 regions, while the alkyne linker, benzoic acid moiety, and *N*-methylpiperazine moiety of ponatinib were all kept intact throughout SAR optimization. The synthesis of the designed derivatives was completed in eight steps. Reductive amination of benzaldehyde derivatives **A–B** yielded benzylpiperazines **68a–b**. Alternatively, selective bromination of toluene derivatives followed by SN2 substitution with 1*N*-methylpiprerazine yielded **68a–c**. Palladium (Pd/C)-catalyzed hydrogenation of nitro aryl intermediates **68a–c** yielded anilines **69a–c**, and coupling with the corresponding iodobenzoic acid derivatives furnished **70a**－**f**. Pd-catalyzed Sonogashira coupling reactions of the commercially available aromatic silyl-protected alkynes or those synthesized *via* Sonogashira reaction conditions from the corresponding bromide precursors with precursors **70a**－**f** and *in situ* removal of the silyl protective group using TBAF furnished **71a**－**t** (Scheme S15).

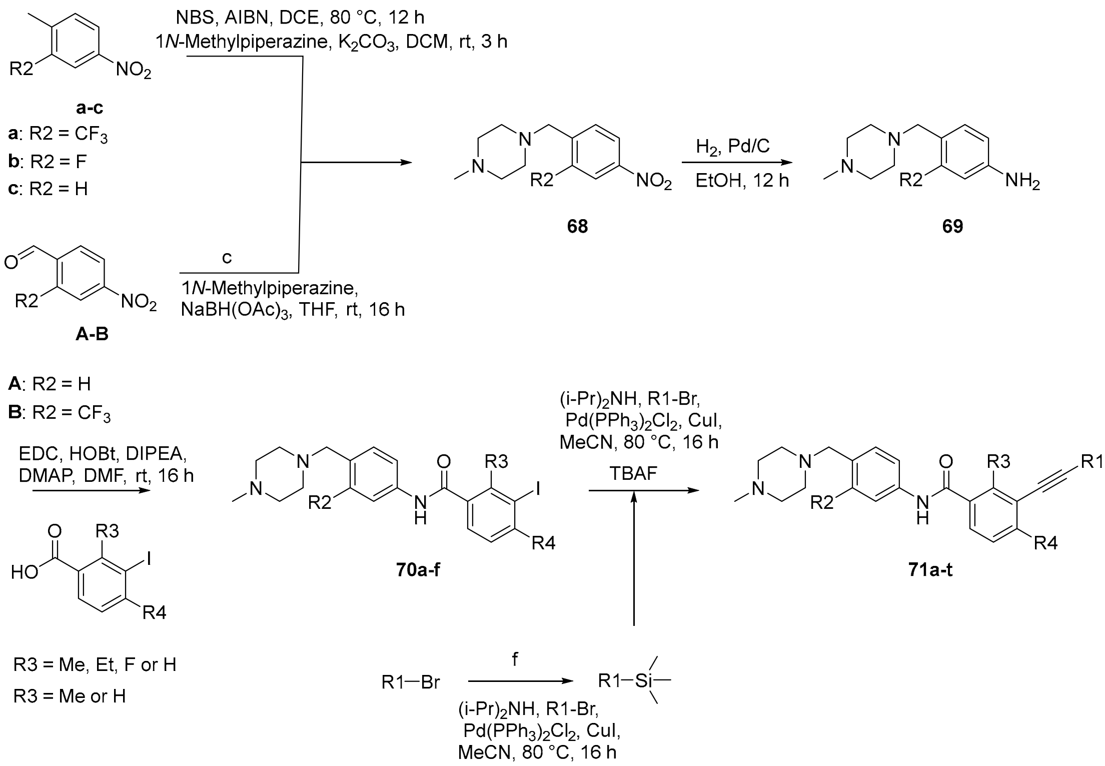

Scheme S15. Synthesis of compounds **70a–d** and **71a**−**t**.

Kettle et al. designed and synthesized a potent derivative and tested it against the KIT mutant Ba/F3 and PDGFR cell lines to treat GISTs [6]. The synthesis of this potent compound was obtained in four steps. Ethyl azidoacetate and 3-methylbut-1-yne underwent a copper-catalyzed cycloaddition to produce the triazole ester **72a**, and the ester group was then hydrolyzed to carboxylic acid **72b**. HATU-mediated amide coupling of the resultant acid **72b** with Boc-protected amine; deprotection of the Boc group then yielded **73b**. Hydroxy alkylation of difluorocyanophenol was followed by the conversion of one F atom to an amine, and the resultant amine **74a** was treated with 1,1-dimethoxy-*N,N* dimethylmethanamine to yield the precursor **74b**. The condensation reaction of **73b** with **74b** in acetic acid yielded **75** (Scheme S16). The synthesis of compounds **AZD2932** and **I-a–f** as quinazoline-based VEGFR and PDGFR inhibitors has been described previously in patent publications [7-10].

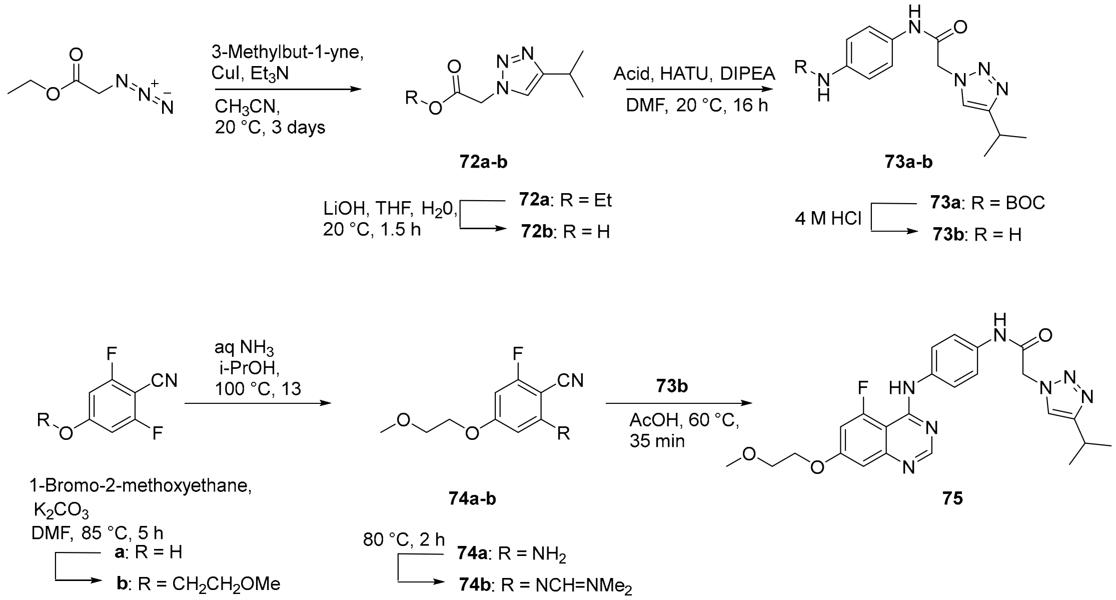

Scheme S16. Synthesis of compound 75.

Wu et al. designed and synthesized a series of 5-phenyl-thiazol-2-ylamine derivatives and screened them for their inhibitory activity for GISTs using c-KIT kinase and GIST-T1 cell lines [11]. Intermediates **76a–b**, **78**, and **78a** were prepared according to established methods [12-14]. Coupling of pyrimidine **80** with the corresponding ester formed urea derivatives **81a–c** and **81g–k**. Treatment of pyrimidine **80** with isocyanate, sulfonyl chloride, and acid chloride produced urea **81d**, sulfonamide **81e,[9]** and amide **81f**, respectively (Scheme S17). Thiazol-2-ylamine **85** was then used to produce the 4-monosubstituted pyrimidine derivative **86** by reacting with 4,6-dichloropyrimidines **77** and NaH in THF, which were then transformed into 4,6-disubstituted pyrimidines **87** using 1-ethylpiperazine in pyridine at 80℃. The generation of 5-iodothiazol-2-ylamine **88** *via* electrophilic iodination followed by Sonogashira coupling of a Boc- or phthalimide-protected alkyne yielded *N*-Boc-protected or phthalimide-protected alkyne intermediates. Deprotection of the Boc group with TFA or phthalimide with hydrazine and subsequent treatment with the respective ester yielded the pyrimidine urea derivatives **90** or **93**. Finally, saturation of the alkyne moiety using Pa d catalyst in MeOH yielded butylamine **94**, which was then treated with ester to yield pyrimidine urea **95** (Scheme S18).

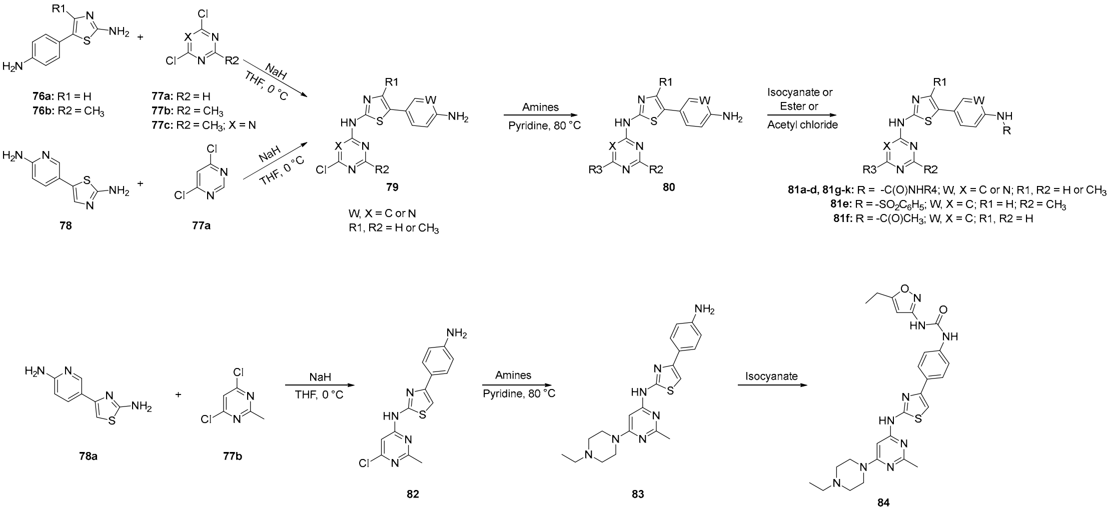

Scheme S17. Synthesis of compounds **81a–k** and **84**.

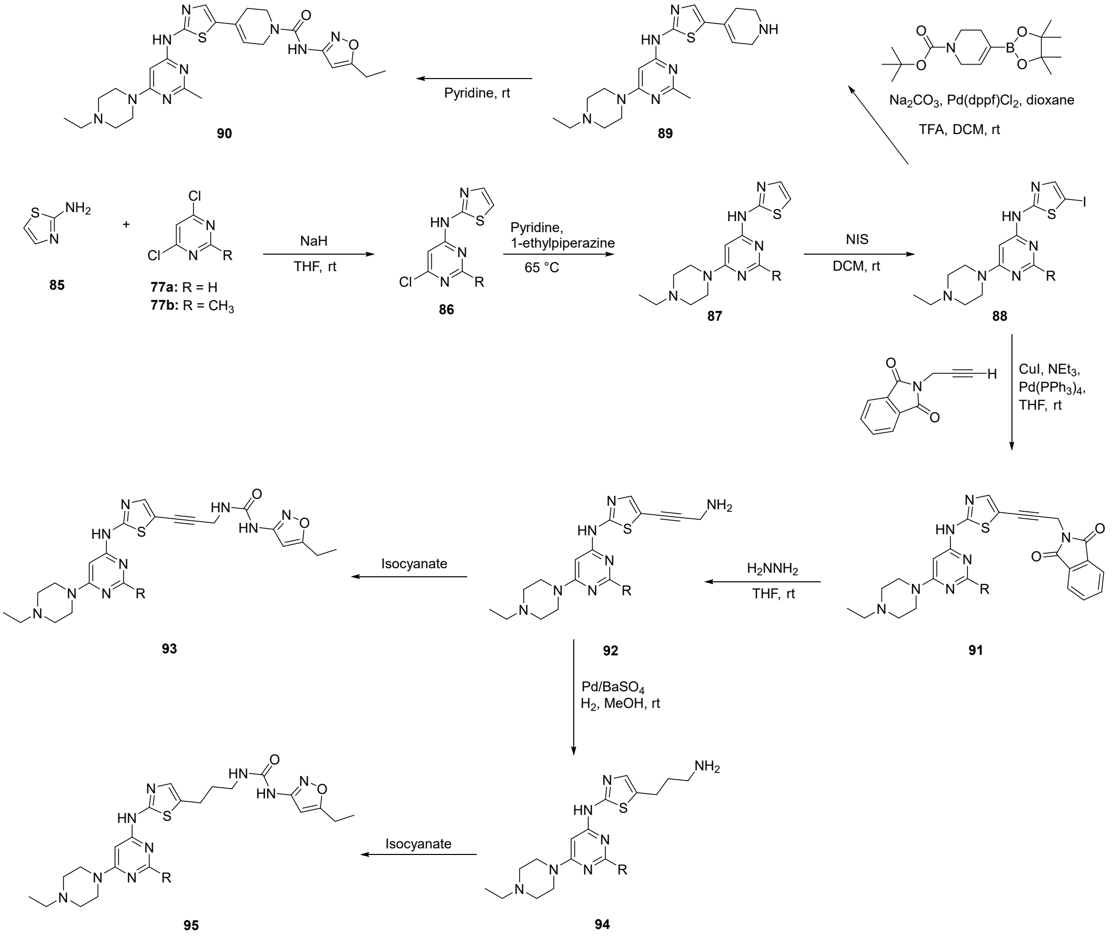

Scheme S18. Synthesis of compounds **90**, **93**, and **95**.

Lin et al. continued their research from their previous compound **81a** by rational design, and the team synthesized a series of 5-aromatic substituted thiazol-2-ylamine pyrimidine derivatives and screened them for their inhibitory activity for GISTs against c-KIT and FLT3 kinases and the GIST-T1 cell line [10]. The process was initiated with the synthesis of 5-aromatic substituted thiazol-2-ylamines **99**, followed by the protection of 2-aminothiazole (**96**) to yield *N*-thiazol-2-yl-propionamide (**97**). Finally, Suzuki coupling of the chloro-substituted pyridine or pyrimidine with **97** yielded 5-aromatic substituted thiazol-2-ylamides **98**. Hydrolysis and neutralization of amide **98** yielded the free 5-aromatic substituted thiazol-2-ylamine **99**, which was used to obtain 4-mono- and 4,6-disubstituted pyrimidine derivatives **101** and **102–106**. The sodium salts of 4-pyridin-3-yl-thiazol-2-ylamine **107** and 1-ethylpiperazine were reacted with 6-chloro and 4-chloro substituents of 4,6-dichloropyrimidine to obtain 4,6-disubstituted pyrimidine **109**, respectively. In addition, amide **111** was obtained by acylating amine **99b** with benzoyl chloride **110** in pyridine. Finally, compounds **102–106**, **105**, and **111** were converted to their corresponding hydrochloride salts by reaction with 6 N HCl in CH_3OH (Scheme S19).

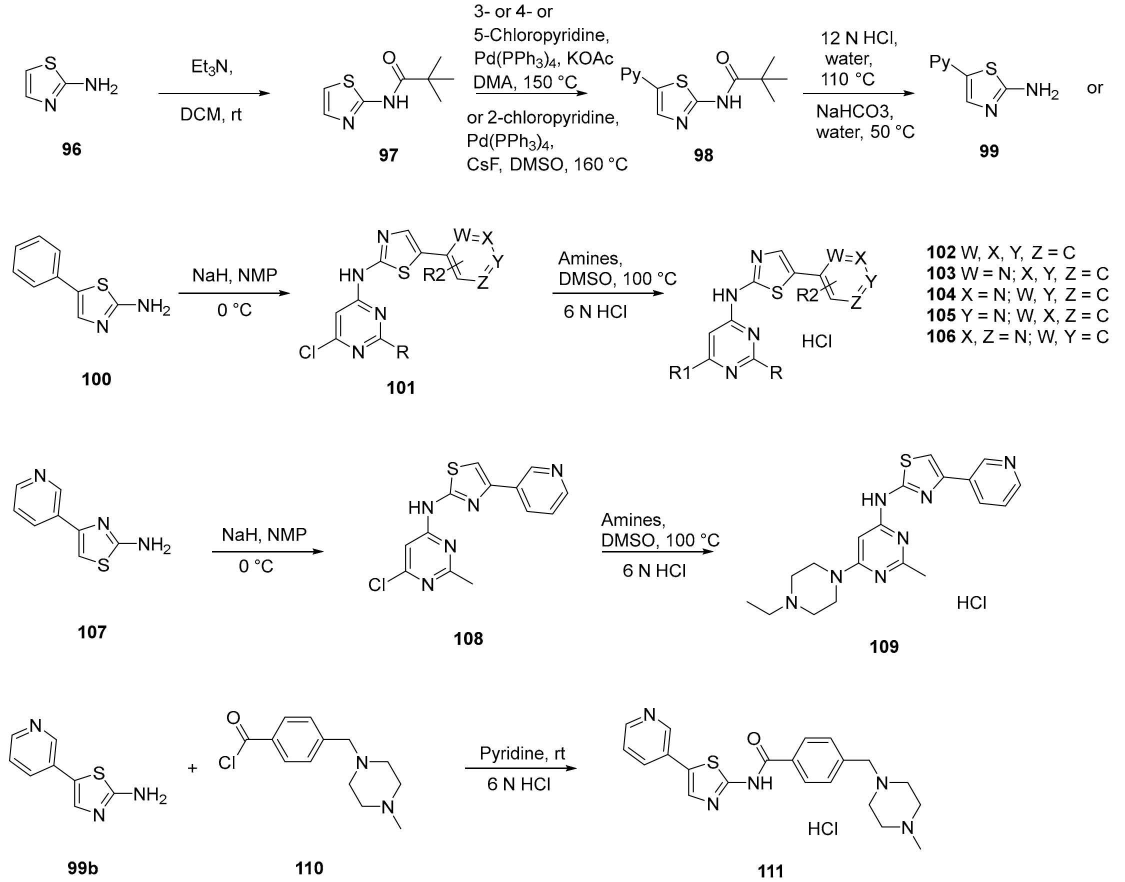

Scheme S19. Synthesis of the designed compounds.

Lu et al. designed structural modifications to linifanib and synthesized a series of 3-methyl-1*H*-pyrazolo[3,4-*b*]pyridine derivatives and screened for their inhibitory activity for GISTs against c-KIT and PDGFRα kinases [15]. The synthesis of the designed compounds was initiated by treating trifluoroacetate with acetonitrile to form 4,4,4-trifluoro-3-oxobutanenitrile (**112**); then, hydrazine-mediated cyclization yielded intermediate **113** (Scheme S20). Multicomponent reaction of **113a** and **113b** with 4-nitrobenzaldehyde and 2,2-dimethyl-1,3-dioxane-4,6-dione followed by reduction of the nitro group

yielded the amine **115**. The amine was reacted with substituted phenyl isocyanates to obtain the desired compounds **116a** and **116b**. Intermediates **117a** and **117b** were obtained by treating **114a** and **114b** with DDQ and BSTFA. Then, reducing the nitro group of **117b** with $SnCl_2$ yielded key intermediate **118**. Target compounds **119a–b** and **112a–n** were then synthesized by reacting substituted isocyanates and isothiocyanates with key intermediates (Scheme S21). The trisubstituted intermediate **123** was generated by Boc protection of **121b**, followed by bromination to form **124**, and hydrolysis to generate **125**. Finally, the target compounds **126a** and **126b** were produced *via* nucleophilic addition of substituted isocyanates. The coupling of various commercially available isocyanates with intermediate **121b** resulted in target compounds **127a–y** (Scheme S22), and intermediate compounds **128a–m** were converted into their respective amines **129a–m** by catalytic hydrogenation. These amines were then coupled with phenyl chloroformate and **121b** to obtain the target compounds **130a–m** (Scheme S23).

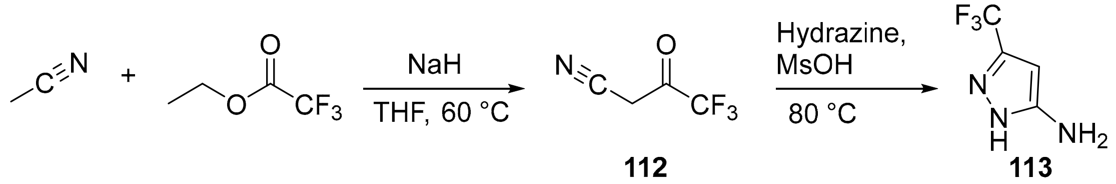

Scheme S20. Synthesis of intermediate **113**.

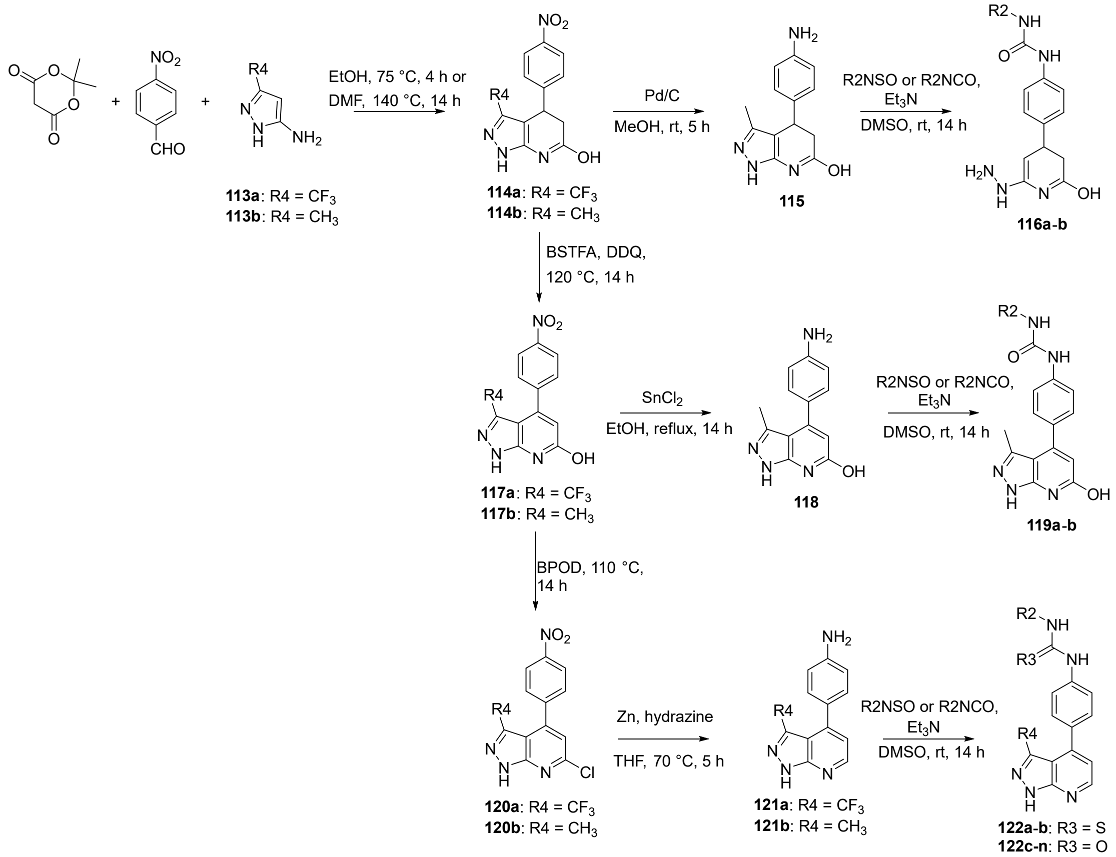

Scheme S21. Synthesis of compounds **116a–b**, **119a–b**, and **122a–n**.

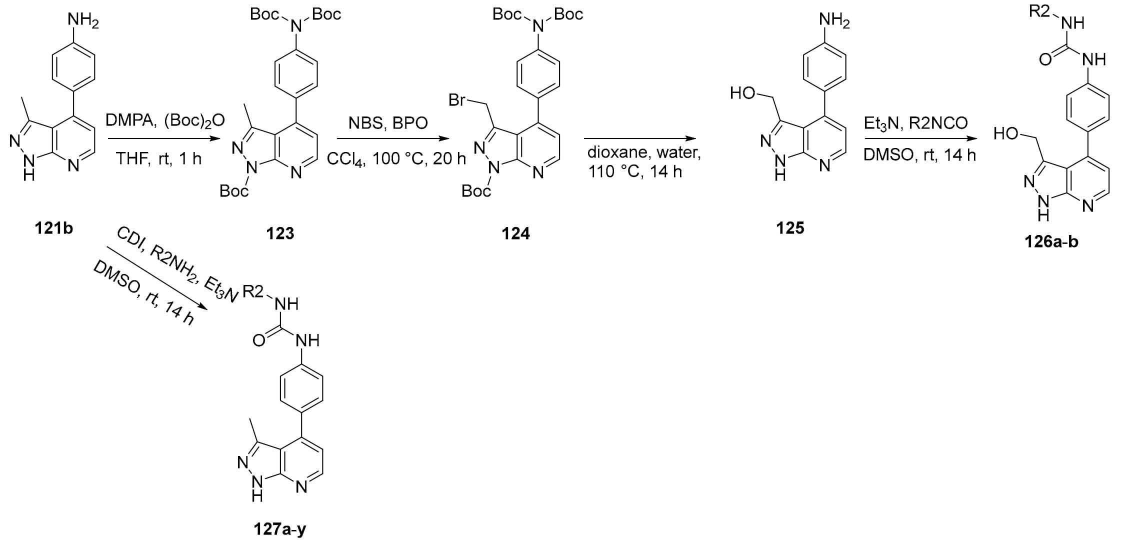

Scheme S22. Synthesis of compounds **126a–b** and **127a–y**.

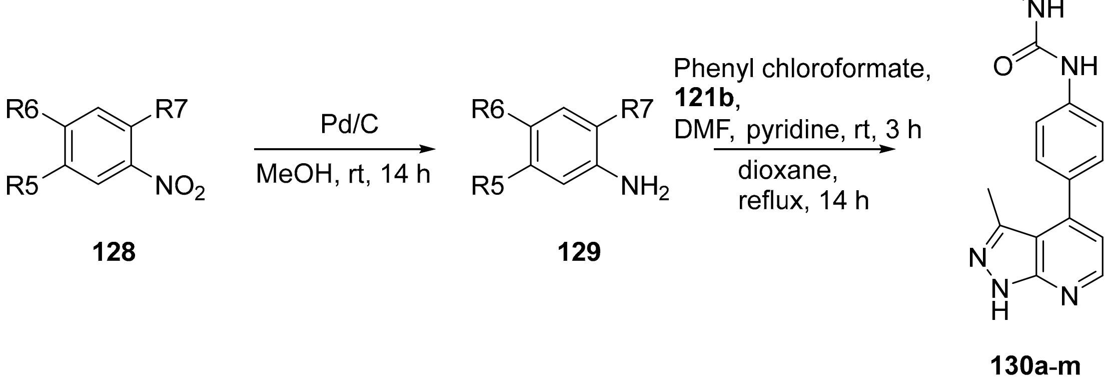

Compd.	R5	R6	R7	Compd.	R5	R6	R7
**128–129a**	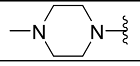	H	H	**128–129h**	H	H	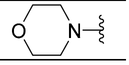
**128–129b**	H_3C–	H	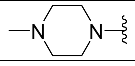	**128–129i**	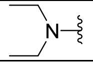	H	H
**128–129c**	H_3C–	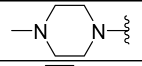	H	**128–129j**	H	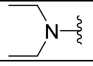	H
**128–129d**	H_3C–	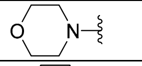	H	**128–129k**	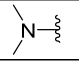	H	H
**128–129e**	H	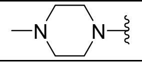	H_3C–	**128–129l**	H	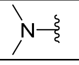	H
**128–129f**	H	H	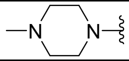	**128–129m**	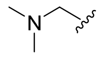	H	H
**128–129g**	H_3C–	H	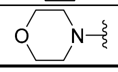				

Scheme S23. Synthesis of compounds **130a–m**.

Andreas et al. designed and synthesized 3-(pyrimidin-4-yl)imidazo[1,2-a]pyridine derivatives as selective c-KIT inhibitors for the treatment of GISTs. The synthesis of the designed targets was completed in three

steps [16]. The common intermediate **131** was obtained by a Suzuki cross-coupling reaction of 4,6-dichloropyrimidine and subsequent condensation with 4-fluoropyridin-2-amine. The SNAr reaction of the chloro group in **131** with various benzylic amines yielded **132**, **133**, and **134**. A second SNAr reaction of the fluoro group with the corresponding alcohols yielded the designed derivatives (Scheme S24).

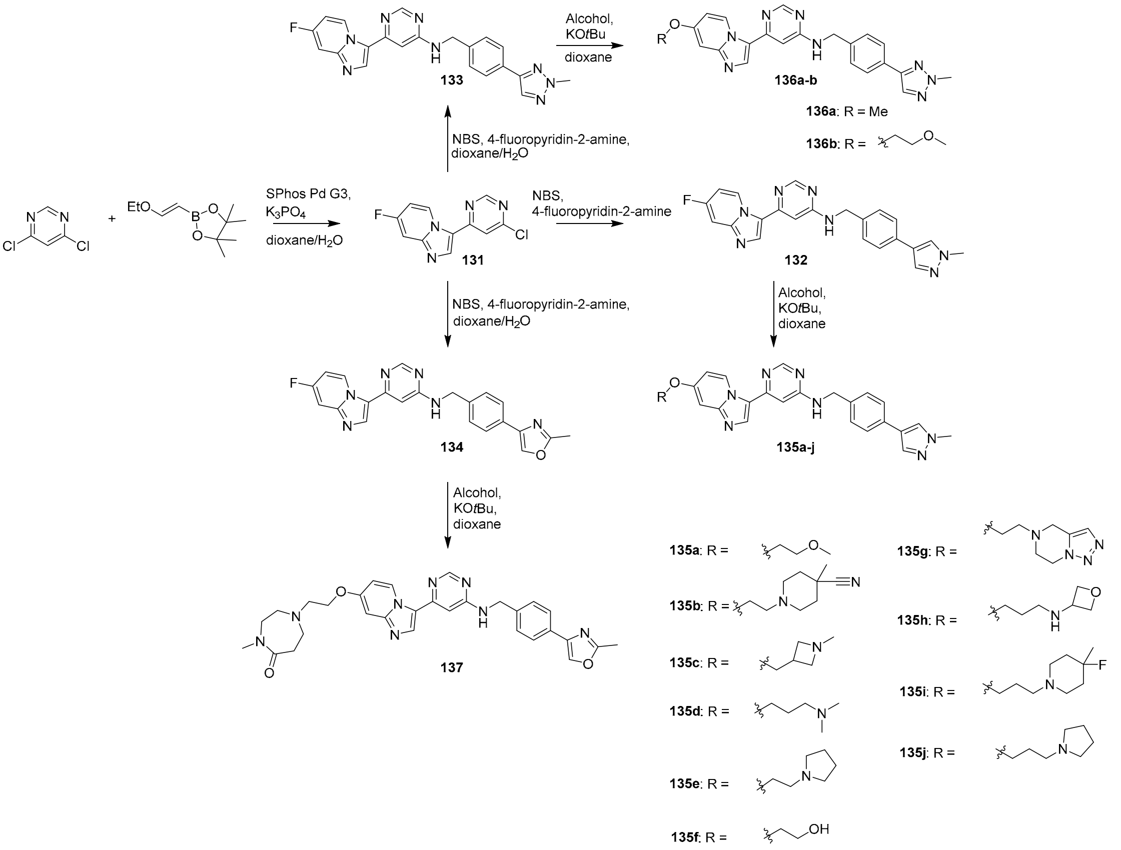

Scheme S24. Synthesis of advanced KIT inhibitors.

Nam et al. designed and reported the synthesis of thiazolo[5,4-*b*]pyridine-based derivatives and screened them for targeting c-KIT kinase and for their antiproliferative activities against GIST-T1 and HMC1.2 cell lines [17]. The synthesis of novel derivatives was completed in six steps. The synthesis started with the formation of aminothiazole, and the amine group was protected with Boc to yield **139**. A Suzuki cross-coupling reaction of **139** with 2-methyl-5-nitrophenylboronic acid yielded **140**. The nitro group of **140** was reduced to an amine group, HATU-mediated amide coupling of the resultant amine with the corresponding carboxylic acid was performed, and removal of Boc provided 1**42a**–**i** and **142k**–**w**. Compound **143a**–**h** amide derivatives were obtained by coupling the amine group with the corresponding acid. The urea derivative **142j** was obtained by treating **141** with the corresponding isocyanate, followed by deprotection of the Boc group with TFA (Scheme S25).

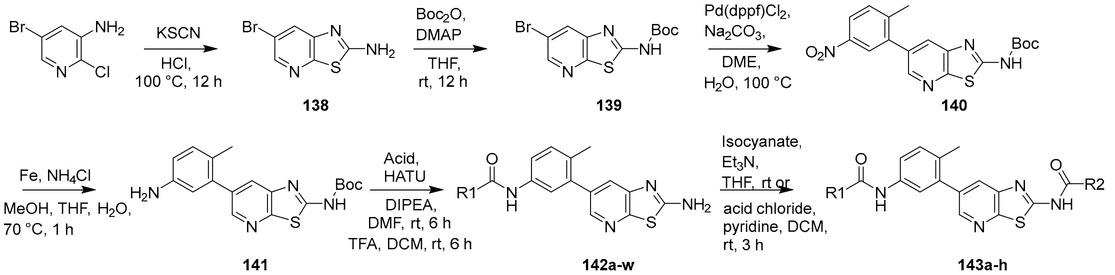

Scheme S25. Synthesis of the designed derivatives **142a–w** and **143a–h**.

References

1. Wang, Qiang, Feiyang Liu, Beilei Wang, Fengming Zou, Cheng Chen, Xiaochuan Liu, Aoli Wang, Shuang Qi, Wenchao Wang, Ziping Qi, Zheng Zhao, Zhenquan Hu, Wei Wang, Li Wang, Shanchun Zhang, Yuexiang Wang, Jing Liu, and Qingsong Liu. "Discovery of N-(3-((1-Isonicotinoylpiperidin-4-Yl)Oxy)-4-Methylphenyl)-3-(Trifluoromethyl)Benzamide (Chmfl-Kit-110) as a Selective, Potent, and Orally Available Type Ii C-Kit Kinase Inhibitor for Gastrointestinal Stromal Tumors (Gists)." *Journal of Medicinal Chemistry* 59, no. 8 (2016): 3964-79.
2. Li, Binhua, Aoli Wang, Juan Liu, Ziping Qi, Xiaochuan Liu, Kailin Yu, Hong Wu, Cheng Chen, Chen Hu, Wenchao Wang, Jiaxin Wu, Zhenquan Hu, Ling Ye, Fengming Zou, Feiyang Liu, Beilei Wang, Li Wang, Tao Ren, Shaojuan Zhang, Mingfeng Bai, Shanchun Zhang, Jing Liu, and Qingsong Liu. "Discovery of N-((1-(4-(3-(3-((6,7-Dimethoxyquinolin-3-Yl)Oxy)Phenyl)Ureido)-2-(Trifluoromethyl)Phenyl)Piperidin-4-Yl)Methyl)Propionamide (Chmfl-Kit-8140) as a Highly Potent Type Ii Inhibitor Capable of Inhibiting the T670i "Gatekeeper" Mutant of Ckit Kinase." *Journal of Medicinal Chemistry* 59, no. 18 (2016): 8456-72.
3. Wu, Yun, Beilei Wang, Junjie Wang, Shuang Qi, Fengming Zou, Ziping Qi, Feiyang Liu, Qingwang Liu, Cheng Chen, Chen Hu, Zhenquan Hu, Aoli Wang, Li Wang, Wenchao Wang, Tao Ren, Yujiao Cai, Mingfeng Bai, Qingsong Liu, and Jing Liu. "Discovery of 2-(4-Chloro-3-(Trifluoromethyl)Phenyl)-N-(4-((6,7-Dimethoxyquinolin-4-Yl)Oxy)Phenyl)Acetamide (Chmfl-Kit-64) as a Novel Orally Available Potent Inhibitor against Broad-Spectrum Mutants of C-Kit Kinase for Gastrointestinal Stromal Tumors." *Journal of Medicinal Chemistry* 62, no. 13 (2019): 6083-101.
4. Liu, Xuesong, Beilei Wang, Cheng Chen, Ziping Qi, Fengming Zou, Junjie Wang, Chen Hu, Aoli Wang, Juan Ge, Qingwang Liu, Kailin Yu, Zhenquan Hu, Zongru Jiang, Wei Wang, Li Wang, Wenchao Wang, Tao Ren, Mingfeng Bai, Qingsong Liu, and Jing Liu. "Discovery of (E)-N1-(3-Fluorophenyl)-N3-(3-(2-(Pyridin-2-Yl)Vinyl)-1h-Indazol-6-Yl)Malonamide (Chmfl-Kit-033) as a Novel C-Kit T670i Mutant Selective Kinase Inhibitor for Gastrointestinal Stromal Tumors (Gists)." *Journal of Medicinal Chemistry* 62, no. 10 (2019): 5006-24.
5. Kaitsiotou, Helena, Marina Keul, Julia Hardick, Thomas Mühlenberg, Julia Ketzer, Christiane Ehrt, Jasmin Krüll, Federico Medda, Oliver Koch, Fabrizio Giordanetto, Sebastian Bauer, and Daniel Rauh. "Inhibitors to Overcome Secondary Mutations in the Stem Cell Factor Receptor Kit." *Journal of Medicinal Chemistry* 60, no. 21 (2017): 8801-15.
6. Kettle, Jason G., Rana Anjum, Evan Barry, Deepa Bhavsar, Crystal Brown, Scott Boyd, Andrew Campbell, Kristin Goldberg, Michael Grondine, Sylvie Guichard, Christopher J. Hardy, Tom Hunt, Rhys D. O. Jones, Xiuwei Li, Olga Moleva, Derek Ogg, Ross C. Overman, Martin J. Packer, Stuart Pearson, Marianne Schimpl, Wenlin Shao, Aaron Smith, James M. Smith, Darren Stead, Steve Stokes, Michael Tucker, and Yang Ye. "Discovery of N-(4-{[5-Fluoro-7-(2-Methoxyethoxy)Quinazolin-4-Yl]Amino}Phenyl)-2-[4-(Propan-2-Yl)-1h-1,2,3-Triazol-1-Yl]Acetamide (Azd3229), a Potent Pan-Kit Mutant Inhibitor for the Treatment of Gastrointestinal Stromal Tumors." *Journal of Medicinal Chemistry* 61, no. 19 (2018): 8797-810.
7. Jung, F. H.; Morgentin, R. R.; Ple, P. "Quinoline Derivatives." *PCT Int. Appl. WO2007099323, 2007.*
8. Jung, F. H.; Ple, P. "Quinazoline Derivatives." *PCT Int. Appl. WO2007099317, 2007.*
9. Chen, Chiung-Tong, John T. A. Hsu, Wen-Hsing Lin, Cheng-Tai Lu, Shih-Chieh Yen, Tsu Hsu, Yu-Ling Huang, Jen-Shin Song, Chun-Hwa Chen, Ling-Hui Chou, Kuei-Jung Yen, Ching-Ping Chen, Po-Chu Kuo,

Chen-Lung Huang, H. Eugene Liu, Yu-Sheng Chao, Teng-Kuang Yeh, and Weir-Torn Jiaang. "Identification of a Potent 5-Phenyl-Thiazol-2-Ylamine-Based Inhibitor of Flt3 with Activity against Drug Resistance-Conferring Point Mutations." *European Journal of Medicinal Chemistry* 100 (2015): 151-61.

10. Lin, Wen-Hsing, Su-Ying Wu, Teng-Kuang Yeh, Chiung-Tong Chen, Jen-Shin Song, Hui-Yi Shiao, Ching-Chuan Kuo, Tsu Hsu, Cheng-Tai Lu, Pei-Chen Wang, Tsung-Sheng Wu, Yi-Hui Peng, Hui-You Lin, Ching-Ping Chen, Ya-Ling Weng, Fang-Chun Kung, Mine-Hsine Wu, Yu-Chieh Su, Kuo-Wei Huang, Ling-Hui Chou, Ching-Cheng Hsueh, Kuei-Jung Yen, Po-Chu Kuo, Chen-Lung Huang, Li-Tzong Chen, Chuan Shih, Hui-Jen Tsai, and Weir-Torn Jiaang. "Identification of a Multitargeted Tyrosine Kinase Inhibitor for the Treatment of Gastrointestinal Stromal Tumors and Acute Myeloid Leukemia." *Journal of Medicinal Chemistry* 62, no. 24 (2019): 11135-50.

11. Wu, Tsung-Sheng, Wen-Hsing Lin, Hui-Jen Tsai, Ching-Cheng Hsueh, Tsu Hsu, Pei-Chen Wang, Hui-You Lin, Yi-Hui Peng, Cheng-Tai Lu, Lung-Chun Lee, Chih-Hsiang Tu, Fang-Chun Kung, Hui-Yi Shiao, Teng-Kuang Yeh, Jen-Shin Song, Jia-Yu Chang, Yu-Chieh Su, Li-Tzong Chen, Chiung-Tong Chen, Weir-Torn Jiaang, and Su-Ying Wu. "Discovery of Conformational Control Inhibitors Switching Off the Activated C-Kit and Targeting a Broad Range of Clinically Relevant C-Kit Mutants." *Journal of Medicinal Chemistry* 62, no. 8 (2019): 3940-57.

12. Lee, Boong Won, and Seung Dal Lee. "[5,5] Sigmatropic Shift of N-Phenyl-N′-(2-Thiazolyl)Hydrazines and N,N′-Bis(2-Thiazolyl)Hydrazines into 2-Amino-5-(P-Aminophenyl)Thiazoles and 5,5′-Bis(2-Aminothiazole) Derivatives†Dedicated to Professor T. H. Chan on the Occasion of His 60th Birthday.†." *Tetrahedron Letters* 41, no. 20 (2000): 3883-86.

13. Décor, Anne, Chantal Grand-Maître, Oliver Hucke, Jeff O◎Meara, Cyrille Kuhn, Léa Constantineau Forget, Christian Brochu, Eric Malenfant, Mégan Bertrand-Laperle, Josée Bordeleau, Elise Ghiro, Marc Pesant, Gulrez Fazal, Vida Gorys, Michael Little, Colette Boucher, Sylvain Bordeleau, Pascal Turcotte, Tim Guo, Michel Garneau, Catherine Spickler, and Annick Gauthier. "Design, Synthesis and Biological Evaluation of Novel Aminothiazoles as Antiviral Compounds Acting against Human Rhinovirus." *Bioorganic & Medicinal Chemistry Letters* 23, no. 13 (2013): 3841-47.

14. Cáceres-Castillo, David, Rubén M. Carballo, Jorge A. Tzec-Interián, and Gonzalo J. Mena-Rejón. "Solvent-Free Synthesis of 2-Amino-4-Arylthiazoles under Microwave Irradiation." *Tetrahedron Letters* 53, no. 30 (2012): 3934-36.

15. Lu, Yanli, Fei Mao, Xiaokang Li, Xinyu Zheng, Manjiong Wang, Qing Xu, Jin Zhu, and Jian Li. "Discovery of Potent, Selective Stem Cell Factor Receptor/Platelet Derived Growth Factor Receptor Alpha (C-Kit/Pdgfrα) Dual Inhibitor for the Treatment of Imatinib-Resistant Gastrointestinal Stromal Tumors (Gists)." *Journal of Medicinal Chemistry* 60, no. 12 (2017): 5099-119.

16. Blum, Andreas, Dieter Dorsch, Nina Linde, Susanne Brandstetter, Hans-Peter Buchstaller, Michael Busch, Nina Glaser, Ulrich Grädler, Aaron Ruff, Carl Petersson, Hanno Schieferstein, Eva Sherbetjian, and Christina Esdar. "Identification of M4205─a Highly Selective Inhibitor of Kit Mutations for Treatment of Unresectable Metastatic or Recurrent Gastrointestinal Stromal Tumors." *Journal of Medicinal Chemistry* 66, no. 4 (2023): 2386-95.

17. Nam, Yunju, Chan Kim, Junghee Han, SeongShick Ryu, Hanna Cho, Chiman Song, Nam Doo Kim, Namkyoung Kim, and Taebo Sim. "Identification of Thiazolo[5,4-B]Pyridine Derivatives as C-Kit Inhibitors for Overcoming Imatinib Resistance." *Cancers* 15, no. 1 (2023): 143.
